# Effects of different exercise types on craving in substance use disorder patients with drug dependence -network meta-analysis and dose-response relationships based on frequentist and Bayesian models

**DOI:** 10.1186/s13722-025-00639-x

**Published:** 2025-12-18

**Authors:** Chuanqiushui Wang, Yi Yang, Kun Wang, Liang Sun, Shi qi Liu, Jiong Luo

**Affiliations:** 1 School of Physical Education, Sports Rehabilitation Center, Southwest University, Chongqing, China; 2 College of Physical Education, Southwest University, Beibei District, Chongqing, China

**Keywords:** Exercise intervention, Substance use disorder, Craving, Dose-response model, Meta-analysis

## Abstract

**Background:**

Exercise interventions have been shown to effectively reduce drug craving and improve physical and mental health in patients with substance use disorders (SUDs). However, the optimal type and amount of exercise needed to maximize these benefits for SUDs is not fully understood and warrants further investigation.

**Methods:**

A comprehensive search strategy was implemented in four electronic databases (i.e., PubMed, Web of Science, CNKI, and EMBASE) to identify randomized controlled trials examining the impact of exercise on craving in individuals with substance use disorders. Network meta-analysis and dose-response modeling were employed to assess the specific benefits of exercise on craving.

**Results:**

The analysis incorporated a total of 30 randomized controlled trials, encompassing a total of 1,717 subjects. These subjects were comprised of 1,258 male participants (73.26%) and 459 female participants (26.73%). The results of the meta-analysis demonstrated that there was a low grade GRADE evidence suggesting that, in comparison with the control group, aerobic exercise (SMD= -0.73, 95%CI: -1.06 to -0.41), high-intensity interval exercise (SMD= -2.19, 95%CI: -3.90 to -0.49), and aerobic combined with resistance exercise (SMD= -1.96, 95%CI: -2.92 to -1.00) were more effective than the control group. Subgroup analyses revealed positive effects of acute aerobic exercise (SMD= -0.23, 95%CI: -0.41 to -0.04, I²=22%) and long-term aerobic exercise (SMD= -0.46, 95%CI: -0.72 to -0.21, I²=0%) on cravings. Furthermore, the results found that Taijiquan significantly reduced drug craving (SMD= -0.47, 95%CI: -0.70 to -0.24, I²=0%) in the subjects. The dosage analysis revealed that the effective range of total exercise for reducing craving in individuals with substance use disorder was from 20 to 320 METs-min/week (SMD= -0.58, 95%CI: -0.8 to -0.28 to SMD= -0.72, 95%CI: -0.91 to -0.46). The optimal form of exercise was determined to be aerobic exercise, with an optimal exercise dose of 180 METs-min/week, which resulted in an estimated mean difference of -1.46 (95%CI: -2.04 to -0.96). The regression analysis results indicated that the impact of exercise on subjects’ cravings may be influenced by their age level (β= -0.995, 95%CI: -2.002 to -0.011).

**Conclusion:**

Aerobic exercise has been recognized as the most effective form of exercise for alleviating drug cravings in individuals with substance use disorders (SUDs). Research indicates that the exercise dose for SUDs exhibits characteristics of low-dose effectiveness and plateaus in its effects. The optimal total intervention dose is best sustained at 180 METs-min/week, which is equivalent to three 60-minute sessions of moderate-intensity aerobic exercise each week.

**Clinical trial registration details:**

Not applicable.

**PROSPERO registration details:**

CRD420251004497.

**Supplementary Information:**

The online version contains supplementary material available at 10.1186/s13722-025-00639-x.

## Highlight

Exercise positively influences drug cravings in individuals with substance use disorders, but this effect is not linear. There exists a non-linear dose-response relationship, which suggests that there may be a “plateau” effect regarding the benefits of exercise on cravings.

Among different types of exercise, aerobic exercise and aerobic combined with resistance exercise have a significant positive effect on craving in people with substance use disorders, and high-intensity interval exercise shows a potentially positive effect.

The best exercise modality for improving craving in people with substance use disorders is aerobic exercise, and the optimal exercise dose is 180 METs-min/week (MD = -1.46, 95% CI: -2.04 to -0.96). The recommended exercise regimen is three 60-minute sessions per week.

**Supplementary Information:**

The online version contains supplementary material available at 10.1186/s13722-025-00639-x.

## Introduction

Presently, the abuse of psychostimulants and related drugs is intensifying worldwide, thereby developing into a global public health crisis. This crisis is causing multidimensional health damage to people with substance use disorders [1, 2]. Physiological research indicates that long-term drug dependence may lead to dysfunction of the dopaminergic system, subsequently triggering abnormalities in the basal ganglia and prefrontal cortex, manifesting as motor impairments and diminished cognitive control abilities [3–5]. Opioids have been shown to markedly elevate the probability of sudden death in individuals with substance use disorder by impeding the function of the respiratory nervous system. In contrast, amphetamine-type stimulants have the potential to induce irreparable damage to the cardiovascular system, thereby becoming the primary cause of mortality among users [6–8]. Among the psychological dimensions associated with patients enduring substance use disorders, it is common for individuals to experience anxiety, depression, and post-traumatic stress disorder. Collectively, these conditions contribute to a detrimental cycle known as “drug-psychological co-morbidity“ [9, 10]. Presently, the mortality rate from opioid overdose continues to increase on a global scale. Additionally, there has been a substantial increase in the use of synthetic drugs, such as methamphetamine, among young people. The clandestine nature and high addictive potential of novel psychoactive substances further complicate prevention and control efforts [11, 12]. While the administration of pharmaceutical remedies in conjunction with cognitive behavioral therapy has been demonstrated to alleviate certain symptoms associated with withdrawal, a significant proportion of individuals exhibit a recurrence rate of symptoms that ranges from 60% to 80% [13–16]. This finding underscores the deficiencies in the efficacy of existing intervention methods.

Craving in the field of addiction is described as a subjective, intense, and irresistible desire or urge to use a substance of abuse, essentially a vicious cycle involving impaired brain reward system function and recruitment of the anti-reward system, leading to compulsive drug use [17]. The corresponding measure, Craving degree, is widely regarded as a pivotal indicator of patients with substance use disorders, and studies have demonstrated a robust correlation between craving degree and the likelihood of relapse [18]. Consequently, within the domain of sports human science, the examination of the impact of exercise on the craving of individuals with substance use disorders holds significant scientific value and clinical translation potential. A number of emerging studies have demonstrated that regular exercise may alleviate withdrawal symptoms by regulating the dopaminergic system and inhibiting inflammatory responses. However, the clinical translation of these findings remains constrained by individual differences in response and the lack of standardization in exercise dosage [19, 20]. A variety of animal models have demonstrated that treadmill training and resistance training have the capacity to upregulate dopamine D2 receptor expression in the nucleus accumbens, thereby leading to a reduction in cocaine self-administration [21–24]. In the context of human studies, moderate-intensity acute aerobic exercise has been observed to transiently reduce subjective ratings of craving during the acute withdrawal period [25]. A long-term exercise intervention has the potential to improve patients’ executive function, modify impulsive decision-making processes, and, by extension, reduce the probability of relapse [26–28]. In addition, exercise may improve brain inflammation and indirectly improve drug craving in patients with substance use disorders by inhibiting the production of inflammatory factors in the brain (e.g., IL-6, TNF-α) and modulating the HPA axis stress response [29–31]. Exercise interventions are also cost-effective and accessible, making them particularly well-suited for use in resource-constrained settings or among populations that resist conventional therapeutic modalities.

A significant amount of research has examined the effects of exercise on cravings in individuals with substance use disorders. In particular, a landmark two-week, double-blind, randomized controlled trial was conducted to examine the influence of Qigong exercise on craving in patients with cocaine use disorder. The results showed that the Qigong exercise group showed a greater degree of craving reduction compared to the placebo group, providing initial evidence for the feasibility of exercise intervention in ameliorating drug craving [32]. An evaluation conducted at two time points, immediately after the intervention and 50 min later, confirmed that the acute improvement in craving levels following moderate-intensity aerobic exercise also applies to individuals with methamphetamine use disorder [33]. Subsequent studies have persisted in investigating the dose-response association between acute aerobic exercise of varying intensity and methamphetamine craving, underscoring the ancillary benefits of moderate-intensity exercise [34]. Numerous studies have consistently explored the long-term effects of different exercise programs on cravings in individuals with substance use disorders. These studies have found that various exercise regimens can have unique impacts on these individuals [35, 36]. This systematic review aims to assess the specific effects of different types of exercise on craving in individuals with substance use disorders. In addition, it aims to predict the potential dose-response relationship between exercise and craving using a dose-response model, ultimately identifying the most effective exercise modality and dosage that can alleviate craving in this patient population.

## Methods

This research has been pre-registered with PROSPERO (CRD420251004497). The methods described in this article comply with the latest PERSiST guidelines and the Cochrane Handbook [37–39].

### Information sources

A comprehensive search strategy was employed, integrating Boolean logic with keywords and synonym replacements to maximize the sensitivity of the search. The Medical Subject Headings (MeSH) keywords included (“drug” or “substance use disorder” or “cannabis” or “cocaine” or “methamphetamine”) and (“exercise” or “training” or “physical activity” or “sports”) and (“craving” or “VAS”). (For a detailed description of the search strategy, refer to the supplementary material) The search was designed to identify research literature published up to February 2025.

### Eligibility criteria

Inclusion criteria: (1) Subjects must have an exercise intervention (e.g., aerobic exercise, resistance exercise, virtual exercise games, or traditional Chinese exercise) included in the intervention program, which includes both single-session and long-term exercise interventions; (2) The studies included in this analysis consist of randomized controlled trials, with the addition of low-quality crossover trials, which have been explicitly addressed in the risk assessment; (3) the study includes a blank control group or a placebo exercise group; (4) Outcome indicators must include assessment of craving; (5) Incorporation of exercise at all intensities.

Exclusion criteria: (1) non-randomized controlled trials; (2) Significant differences in baseline data between subgroups of study participants and unavailability of suitable data; (3) exclude dissertations, conference papers, non-human experiments, and books.

### Data processing

Two reviewers, C.W. and Y.Y., independently extracted relevant data from the studies that met the inclusion criteria, and disagreements were resolved by a third evaluator, J.L. When available, the mean and standard deviation were directly obtained from the articles [40]. The extracted data encompasses the first author, publication year, demographic characteristics, intervention model, level of craving, and other pertinent outcome indicators. If data from a research article is unavailable, an official email will be sent to the corresponding author to request the necessary information.

This study employed metabolic equivalents of task (MET) to quantify exercise dosage. Intensity per unit of exercise is defined as follows: intensity of a specific exercise pattern (MET) x duration of a single exercise session (excluding warm-ups and intervals) x number of exercises per week [41, 42]. The resultant data were then coded according to the equivalents specified in the “2011 Compendium of Physical Activities” [43]. In instances where the exercise durations were vague or subject to change, the mean value was estimated. The frequency of exercise conducted weekly was quantified by the total number of exercise interventions administered during that period. The aggregate volume of these exercise interventions was reported in terms of METs-mins/weeks [44, 45]. The categories of exercise interventions were delineated employing the consolidated definitions from extant studies and subsequently classified into the following: aerobic exercise, resistance exercise, traditional Chinese exercises, combined aerobic and resistance exercises, high-intensity interval exercises, and miscellaneous exercises. (See Table 1 for details.)

**Table 1:** Classification of exercise intervention types

Exercise type	Definitions (inclusive)
Aerobic exercise(AE)	Continuous exercise that consistently improves an exerciser’s cardiorespiratory fitness based on the heart rate division during exercise, including running, walking, cycling, etc.
Resistance exercise(RE)	Exercises based on improving an exerciser’s muscle mass, strength endurance, or strength function, including equipment resistance or self-weighted resistance.
Chinese traditional exercises(CT)	Exercises included in traditional Chinese culture for the purpose of cultivating the body, fitness and strengthening the body include Baduanjin, taijiquan, Qigong, and staking.
Combined aerobic and resistance exercises(ARE)	Training based on the American College of Sports Medicine’s recommended combination of aerobic and resistance exercises, including treadmill, machine resistance, Swiss ball training, etc [46].
Others exercises(OE)	The rest of the sports that may have a positive impact on subjects’ cravings and are actually used include dance, soccer, and circuit training.
High-intensity interval exercises(HIIT)	High-intensity short repetitions based on peak oxygen uptake and maximal heart rate division.

### Study risk of bias assessment

Risk of bias was assessed for the included studies by two assessors (C.W. and Y.Y.) [47]. Given the challenges associated with fully blinding participants in exercise intervention programs, this meta-analysis offers a comprehensive examination of the associated risks of blinding. For trials employing a blinding design that does not explicitly label the experimenter, the risk of blinding is assessed based on the significance of the discrepancy between the intervention and control group intervention procedures. In instances where the control group involves a placebo exercise design, it is categorized as “low risk” or “unclear risk.” Conversely, if the control group is a blank control group, it is classified as “high risk”. The quality of the evidence for the effects of the interventions in the studies included in this review was assessed using the GRADE approach. Any discrepancies in evaluation were resolved through the involvement of a third evaluator, J.L [48, 49].

### Measurement effect

Statistical analysis was performed with R software. Because of the variability of the outcome measures, standardized mean differences and standard deviations were used to calculate effect sizes to summarize and compare results. This study utilized a random effects model for the meta-analysis, with the combined effect size derived from the SMD of the intervention effect. The resulting effect size was reported along with a 95% confidence interval. Additionally, the Cochrane Q p-value and I² statistics were employed to evaluate heterogeneity among the studies. A study was deemed heterogeneous if the p-value was less than 0.05 and the I² statistic exceeded 50% [50]. Sensitivity analyses were performed using STATA 18.0 software, and the Egger’s test and Begg’s test were used to examine the research results for possible publication bias [51]. In the calculation of the standard deviation difference, the correlation coefficient was designated as *r* = 0.5. This value is deemed acceptable for the repeatability of moderate-level measurements in accordance with prevailing literature.

### Synthesis methods

#### Meta-analysis

The present study utilized a Bayesian model to conduct a pairwise meta-analysis of all incorporated studies, aiming to analyze the actual effects of various exercise interventions in comparison to the control group. Following this, the results of both direct and indirect comparisons of the different exercise interventions on substance craving were evaluated using a frequentist model with standard mean differences. Additionally, interventions with five or more studies were independently analyzed employing a random-effects model, with subgroup analyses initiated based on heterogeneity tests. Data that had been adjusted for bias formed the foundation for reporting effect sizes, ensuring the validity of inferences regarding the moderating impact of exercise interventions on substance craving.

#### Network meta-analysis

The network analysis was executed using a Bayesian model and the network tool Metainsight (version 5.0.1) [52]. The present study yielded a network diagram illustrating the various exercise interventions that were the focus of the evaluation. This network diagram was utilized to visually represent the specific effects of these interventions. A forest plot of the cumulative sum of the ranked area under the curve of different interventions was also generated using the control group as the baseline [53].

#### Dose-response analysis

A dose-response analysis was performed using R software. A Bayesian random-effects model was employed to assess the association between exercise dosage and craving. Initially, the data were examined to ascertain that they met the assumptions of a linear dose-response relationship and the MBNMA network (network transitivity, consistency, and connectivity) [54, 55]. Subsequently, a parametric Emax model were constructed and fitted for comparison. This comparison was used to assess the suitability of different dose-response curves and the robustness of the model. Ultimately, the statistical reliability of the exercise dose effect was comprehensively verified [56]. The present study employed a posteriori estimation of the dose-response model, a deviation chart to assess the goodness of model fit, the treats function based on a Bayesian framework to calculate the relative effect and complete the Rank probability ranking, and simultaneously generated the SUCRA curve to cross-validate the results with Metainsight. A prediction curve was further constructed with a placebo dose of 0 METs-min/week, and statistical significance was determined by excluding the zero value through the 95% confidence interval. Concurrently, the potential range of effects of the intervention was delineated based on the 95% prediction interval to facilitate multi-level inference of the dose-effect relationship of exercise [57].

#### Regression analysis

Network regression analysis was employed to investigate the potential impact of moderating variables [58, 59]. The present study operates under the assumption that all interventions possess a therapeutic effect. The year of addiction, gender ratio, age, year of abstinence, body weight, intervention duration, sample size, and intervention intensity were considered as potential influencing variables. The exercise intervention cycle is expressed in weeks, and studies of single acute interventions are uniformly expressed as an intervention cycle of 0.1 weeks in the exercise intervention cycle. The intensity of the intervention was defined based on the heart rate achieved during various exercises in the study. The intervention intensity was categorized into three levels: heart rates below 40% of the maximum were classified as low intensity, those between 40% and 70% were considered moderate intensity, and rates exceeding 70% were designated as high intensity. In cases where heart rate monitoring was unavailable, the study’s established definitions served as the standard. Missing data were subsequently replaced with the mean value. The B coefficient of the model was utilized to estimate the effect size, and a 95% confidence interval that excludes 0 indicates statistical significance.

## Results

### Inclusion of study

**Fig. 1:**
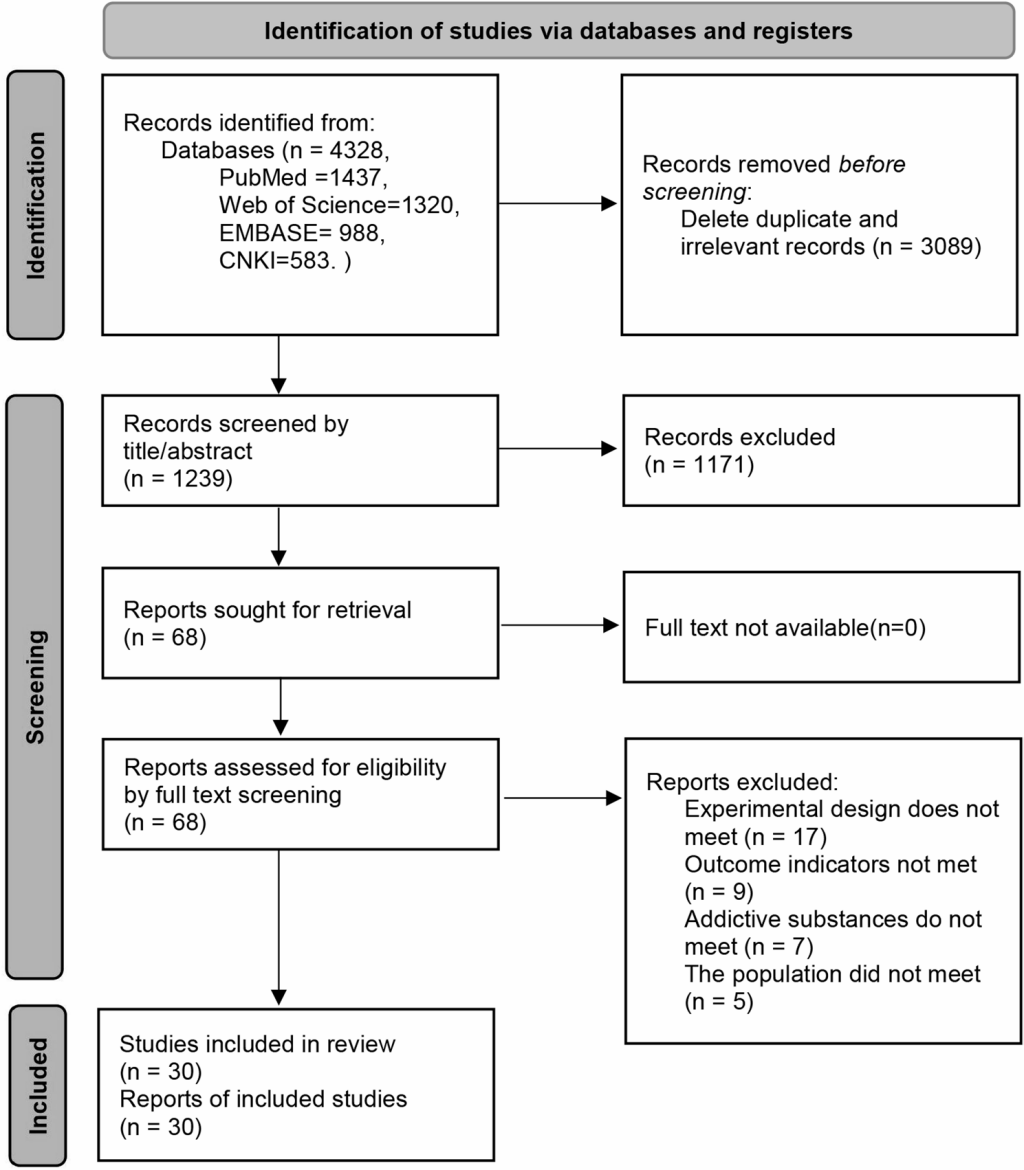
Literature screening flowchart

A comprehensive search strategy yielded a total of 4,328 documents from four distinct databases, as delineated in the PRISMA 2020 flow diagram. Following an initial screening by two researchers, 3,089 duplicate or irrelevant studies were excluded. Subsequently, 1239 studies were read for titles and abstracts, and a further 1171 irrelevant studies were deleted. Subsequently, 68 studies were reviewed in full text, and those that did not meet the inclusion criteria were excluded. The final meta-analysis incorporated a total of 30 articles. The search flowchart is shown in Fig. 1.

### Characteristics of the study

This review encompasses a total of 30 studies that collectively involved 1,717 participants. Among these participants, there were 1,258 men, representing 73.26% of the sample, and 459 women, constituting 26.73%. The mean age of participants within individual studies ranged from 18 to 52. The studies were conducted between 2013 and 2025. The geographical distribution of the studies covered major regions such as China, the United States, Norway, and Brazil. Specifically, five studies were from the United States, one study was from Brazil, two studies were from Norway, and 22 studies were from China. The exercise interventions were classified into the following categories: aerobic exercise (e.g., walking, treadmills, cycling, aerobics), resistance exercise (e.g., dumbbells, strength training), traditional Chinese exercises (e.g., Baduanjin, Taijiquan, standing meditation, Qigong), aerobic combined with resistance exercise, and other exercises (e.g., functional exercises, football, circuit training, dance). The duration of the interventions ranged from immediate interventions to long-term interventions of 12 weeks. The substance use included cocaine, methamphetamine, amphetamine, cannabis, heroin, and ecstasy, among others (refer to supplementary material for a comprehensive list of the included studies).

Secondly, the present study screened the outcome indicators of each included study and evaluated craving as the primary outcome indicator. Of the included studies, 25 employed the visual analog scale (VAS) as an effective indicator to measure the subject’s craving [33–36, 60–81]. Two studies employed the Cocaine Craving Questionnaire Brief as an effective indicator of the subject’s craving [32, 82, 83]. One study employed the Voris Cocaine Craving Scale as an effective indicator of the subject’s craving [32, 84]. One study employed the Marijuana Craving Questionnaire - Short Form as a reliable indicator of the subject’s craving [85, 86]. One study used the amphetamine craving scale as an effective indicator of the subject’s craving [87, 88]. One study used Craving questionnaires-short forms as an effective indicator to measure the subject’s craving [89, 90].

The selection of the remaining outcome indicators in this study incorporated measures of the psychological function of the subjects. However, these measures were not included in the results of the meta-analysis, as this was not the study’s primary objective. Concurrently, other studies employed instruments such as the Patient Health Questionnaire, the Beck Depression Inventory, and the Self Rating Depression Scale to assess the depression of the subjects [32, 62, 65, 71, 73, 80, 88, 89]. Some studies investigated the anxiety of the subjects using the Generalized Anxiety Disorder Survey, Self Rating Anxiety Scale, Spielberger State-Trait Anxiety Inventory-State only, and Hamilton Anxiety Scale [32, 62, 65, 73, 80, 88, 89]. A number of studies have examined the cognitive function of subjects by means of cognitive neuropsychological testing [35, 67, 69, 76, 80].

### Meta-analysis results

The paired meta-analysis included seven intervention types, a total of 42 two-arm comparisons, including 2,382 subjects, and a total of 21 possible paired comparisons. The final random-effects model confirmed a mean effect of -3.59 for exercise interventions on participants’ substance craving. Among all included exercise interventions that had an effect on participants’ substance craving, there were AE (SMD = -0.73, 95% CI: -1.06 to -0.41), ARE (SMD = -1.96, 95% CI: -2.92 to -1.00), HIIT(SMD = -2.19, 95% CI: -3.90 to -0.49), CT (SMD = -0.27, 95% CI: -0.81 to 0.27), RE(SMD = -0.66, 95% CI: -1.47 to 0.16), and OE(SMD = -0.31, 95% CI: -1.45 to 0.84). (For details, see the supplementary material.)

Secondly, this study conducted a comprehensive meta-analysis focused on both aerobic exercise and traditional Chinese exercise, utilizing a large sample size (detailed results can be found in the supplementary material). The findings indicated that traditional Chinese exercise had a significant positive impact on subjects’ substance cravings (SMD = -0.28, 95% CI: -0.50 to -0.07, I² = 38%). Additionally, aerobic exercise interventions also demonstrated a significant positive effect on participants’ substance cravings (SMD = -0.75, 95% CI: -1.03 to -0.46, I² = 81%). A sensitivity analysis was conducted on the aerobic exercise intervention due to the observed high heterogeneity, leading to the exclusion of certain highly heterogeneous studies. Following this, a subgroup analysis was performed on the aerobic exercise intervention based on the modified model. The results revealed the effects of acute aerobic exercise on participants’ substance cravings (SMD = -0.23, 95% CI: -0.41 to -0.04, I² = 22%), as well as the effects of long-term aerobic exercise (SMD = -0.46, 95% CI: -0.72 to -0.21, I² = 0%). The results of the subgroup analysis of traditional Chinese exercise showed that Taijiquan had an effect on the participants’ craving for substances (SMD = -0.47, 95% CI: -0.70 to -0.24, I² = 0%). The effect of Baduanjing on the degree of craving (SMD = -0.03, 95% CI: -0.69 to -0.62). The effect of standing meditation (Chanzhuang) on the subject’s craving for substances (SMD = 0.56, 95%CI: -0.03 to 1.16). The effect of Qigong on the subject’s drug craving (SMD= -0.29, 95%CI: -0.60 to 0.01).

The results of the frequencyist model study give more detailed results for the comparison between different exercise interventions. The results further indicate that ARE may show more positive effects than other exercise interventions. ARE compared with AE (SMD: -1.22, 95% CI: -2.24 to -0.21). ARE compared to RE (SMD: -1.30, 95% CI: -2.56 to -0.04). ARE compared to OE (SMD: -1.65, 95% CI: -3.14 to -0.16). ARE compared with CT (SMD: -1.69, 95%CI: -2.80 to -0.59). HIIT also showed a more positive effect than CT (SMD: -1.93, 95%CI: -3.72 to -0.14) (See Table 2).

**Table 2:**
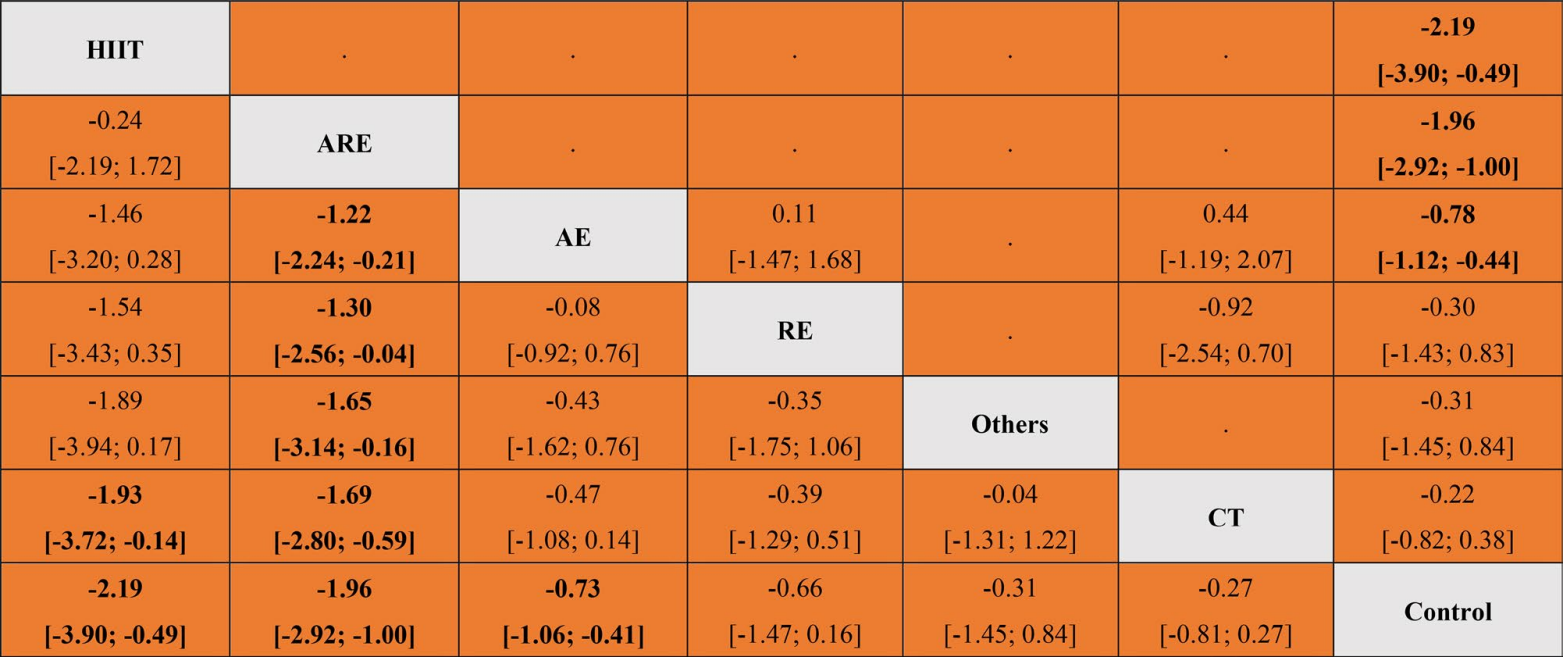
Union table of direct and indirect comparisons of the effects of different interventions on drug craving

### Bayesian network meta-analysis results

**Fig. 2:**
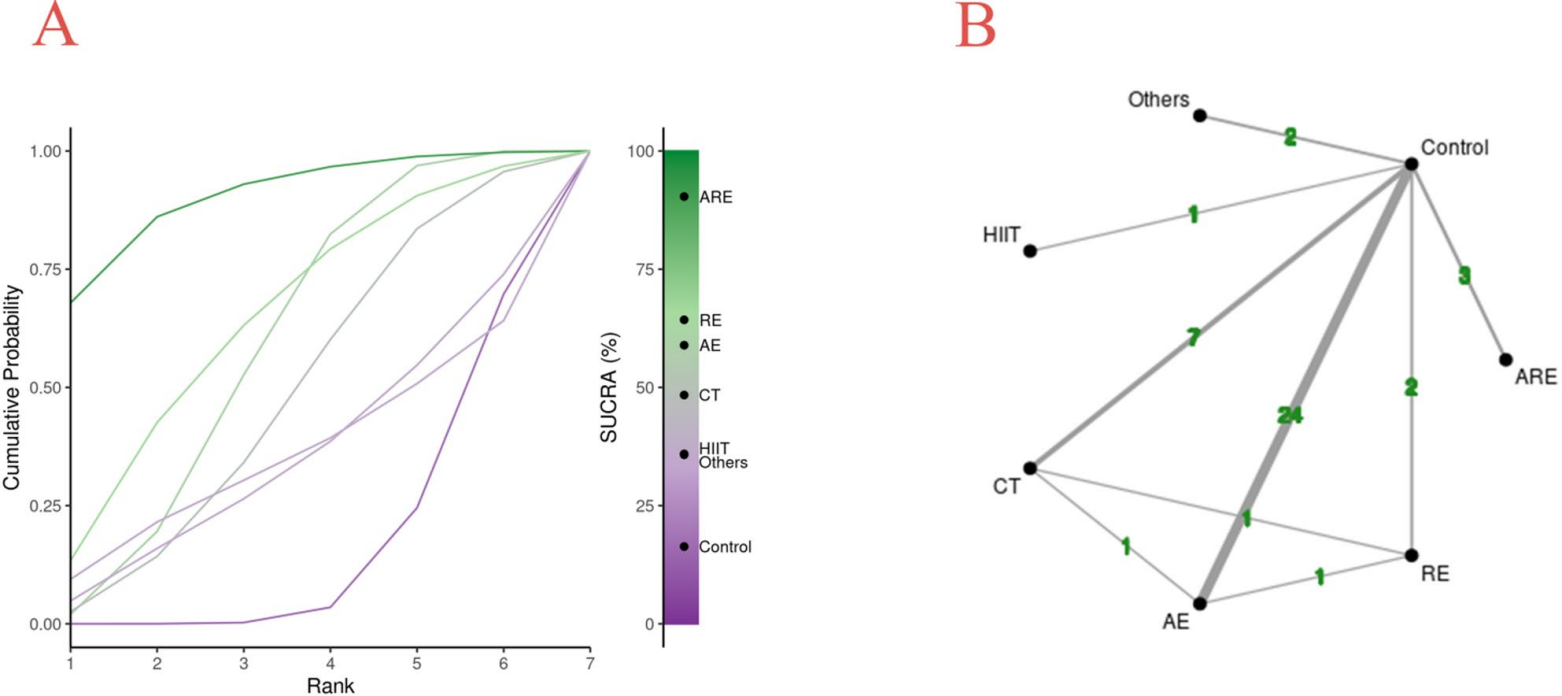
**A**: Bayesian network plot. **B**: Network diagram

Initially, the study utilized the Bayesian network model to assess the connectivity among the exercise interventions under consideration. Subsequent to the confirmation of intervention connectivity, a network diagram was formulated to illustrate the effects of diverse exercise interventions on the subject’s substance craving, encompassing the connections and number of connections between each exercise intervention. (See Fig. 2-A.) The specific results are as follows. The model encompasses seven nodes, which encapsulate twenty-four aerobic exercise versus control group comparison trials, three aerobic combined with resistance exercise versus control group comparison trials, seven traditional Chinese exercises versus control group comparison trials, two resistance exercises versus control group comparison trials, one high-intensity interval exercise versus control group comparison trials, two other exercises versus control group comparison trials, one traditional Chinese exercise versus resistance exercise comparison trial, one aerobic exercise versus resistance exercise comparison trial, and one aerobic exercise and traditional exercise.

A Bayesian network model was used to calculate the SUCRA values of different exercise interventions to evaluate the ranking effect of the impact of exercise interventions on the subject’s substance craving. The results of the ranking effect showed (from high to low) that the SUCRA value of aerobic combined with resistance exercise (ARE) was 90.38%, the SUCRA value of resistance exercise (RE) was 64.30%, the SUCRA value of aerobic exercise (AE) was 58.92%, Traditional Chinese exercise (CT) SUCRA value of 48.38%, high-intensity interval training (HIIT) SUCRA value of 35.93%, other exercises (Others) SUCRA value of 35.75%, control group (Control) SUCRA value of 16.34% (See Fig. 2-B).

### Dose-response analysis results

**Fig. 3:**
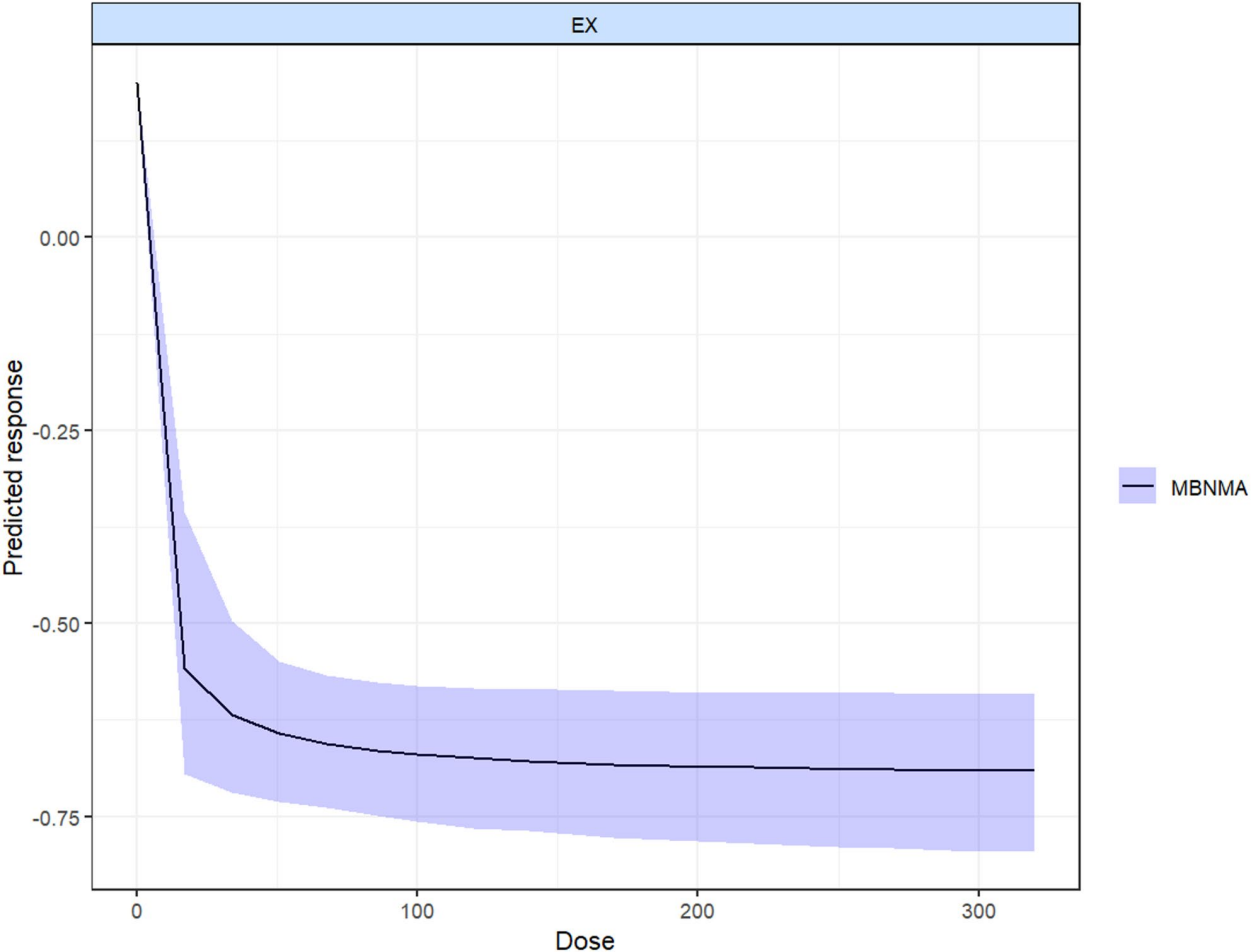
Presents the hypothesized relationship between the total dose of exercise and the subsequent craving, as predicted by the established dose-response model

This study employed a dose-response model to investigate the relationship between the quantity of exercise and the intensity of physical craving in total weekly exercise. The findings revealed a non-linear dose relationship between exercise intervention and physical craving in subjects (see Fig. 3 for specifics). A pronounced increase in craving was observed immediately following the commencement of exercise, coinciding with a period of exercise. The model predicts that the minimum effective exercise dose is 20 METs-min/week (MD= -0.58, 95%CI: -0.8 to -0.28), and the optimal exercise dose occurs at 320 METs-min/week (MD= -0.72, 95%CI: -0.91 to -0.46).

**Fig. 4:**
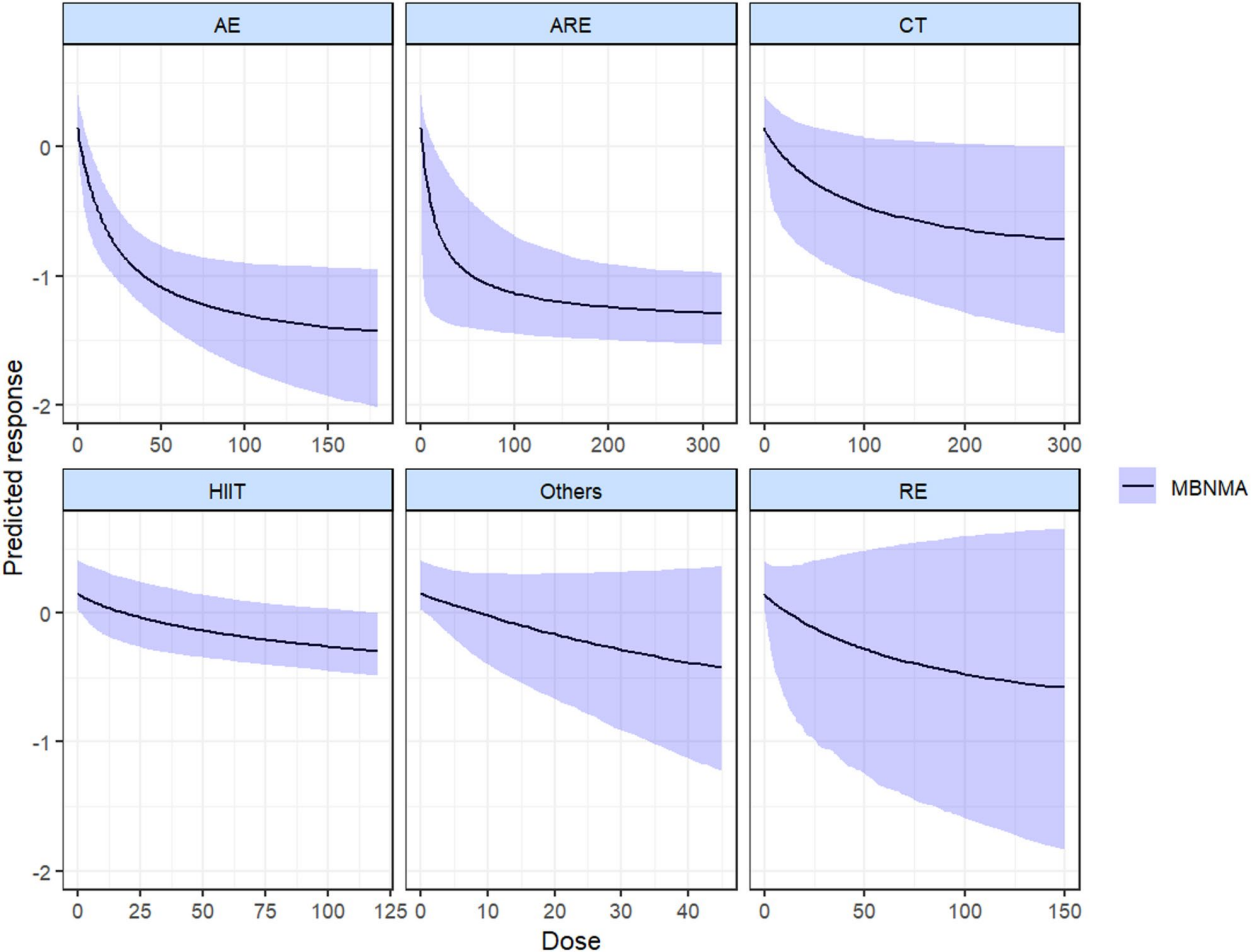
Dose-response prediction curves for the effect of various sports on craving, based on dose-response models

The findings of the analysis of each exercise intervention employing the dose-response prediction model indicated that all six included exercise interventions exhibited a non-linear dose-response relationship with the participants’ substance craving to varying extents, with nearly all the curves demonstrating a negative correlation (see Fig. 4 for specifics). This suggests that the participants’ substance craving diminished with exercise. Significant positive effects were observed in the prediction curve for the aerobic exercise intervention, the aerobic exercise combined with resistance exercise, and the high-intensity interval exercise. The absence of a U-shaped curve in any of the exercise interventions suggests the potential absence of an exercise threshold within a controllable dose. Among the exercise interventions, aerobic exercise exhibited a relatively smooth prediction curve, suggesting a potentially significant reference value. This was mainly due to differences in sample size and other factors. In contrast, the prediction curves for traditional Chinese exercises, high-intensity interval exercises, aerobic combined with resistance exercises, resistance exercises, and other exercises were too scattered.

The subsequent prediction model provides a ranking of the effects of different dose interventions in the model. The results indicate that when a parametric model is used to predict dose interventions, the rankings are as follows: aerobic exercise, aerobic exercise combined with resistance exercise, traditional Chinese exercise, high-intensity interval exercise, other exercise, and resistance exercise. The minimum effective dose of AE intervention was determined to be 9.5 METs-min/week (MD= -0.4, 95%CI: -0.72 to -0.09), and the optimal exercise dose was identified as 180 METs-min/week (MD= -1.46, 95%CI: -2.04 to -0.96). The minimum effective dose of CT intervention was 45 METs-min/week (MD= -0.28, 95%CI: -0.82 to 0.15), and the optimal exercise dose was 300 METs-min/week (MD= -0.71, 95%CI: -1.42 to 0.02). The minimum effective dose for the ARE was determined to be 20 METs-min/week (MD= -0.64, 95%CI: -1.31 to -0.06), and the optimal exercise dose was identified as 320 METs-min/week (MD= -1.28, 95%CI: -1.52 to -0.99). The minimum effective dose for HIIT was determined to be 120 METs-min/week (MD= -0.28, 95%CI: -0.48 to -0.01). No significant positive effect on the prediction curves was observed for OE and RE. (Further elucidation can be found in the supplementary material.)

### Reporting bias and GRADE rating quality

#### Risk of bias and publication bias

In this meta-analysis of 30 studies, 13 were identified as having a high risk of bias. In contrast, 22 studies were categorized as having an indeterminate risk of bias, while five studies were rated as having an exceptionally low risk of bias. Below are some of the reasons for these classifications. One experiment divided low-intensity, moderate-intensity, and high-intensity intervention groups according to exercise intensity. Although the blinding of the study was not mentioned, the risk of unclear blinding was given because the subjects’ perception of exercise intensity may have a placebo effect [34]. One study evaluated the participant blinding and determined it to be at low risk, as the control group incorporated a consistent exercise intervention [79]. In a study where the control group was passively maintained in a seated position, the subjects could easily perceive the situation and determine it as high risk [61].

Due to the limited sample size of different types of exercise, this study examined the risk of publication bias in aerobic exercise and traditional Chinese exercise. The results of the publication bias test for aerobic exercise indicated that certain studies may be susceptible to bias (Egger’s test: P>|t|=0.002, Begg’s test: Pr>|t|= 0.016). Conversely, the results of the publication bias test for traditional Chinese exercises did not indicate any significant publication bias (Egger’s test: P>|t|=0.03, Begg’s test: Pr>|t|= 0.09).(See supplementary material for detailed results).

#### GRADE quality

The quality of the evidence regarding participants’ drug cravings was determined to be low in this study, as assessed by the GRADE quality scale applied to the included studies. For a thorough evaluation of these studies, please consult the supplementary material.

### Regression analysis

The results of the regression analysis are shown in Table 3. The results show a negative relationship between the subject’s year of addiction and the predictions of the model, as indicated by the regression coefficient (β= -0.302, 95%CI: -2.191 to 1.632). The result of the influence of the subject’s female proportion on the model is (β = 0.036, 95%CI: -0.684 to 0.72). The result of the influence of the subject’s age on the model is (β= -0.995, 95%CI: -2.002 to -0.011), which is significant. The final result of the influence of the subject’s addictive substances on the model is (β = 0.569, 95%CI: -0.285 to 1.424), which is significant. The effect of the subject’s exercise intervention cycle on the model was (β= -1.115, 95%CI: -11.6119 to 6.609). The effect of total sample size on the model was (β= -0.684, 95%CI: -1.816 to 0.275). The effect of the intensity of the subject’s exercise intervention on the model was (β = 0.11, 95%CI: -0.697 to 0.924). The specific effects of all covariates are shown in Table 3.

**Table 3:** Meta-regression

Covariate	Shared beta	Heterogeneity standard deviation
-	-	2.01 (1.234 to 2.983)
Addiction year	-0.302 (-2.191 to 1.632)	2.13 (1.457 to 3.029)
Percentage of females	0.036 (-0.684 to 0.72)	1.04 (0.743 to 1.432)
Ages	-0.995 (-2.002 to -0.011)*	0.919 (0.655 to 1.28)
Addictive substances	0.569 (-0.285 to 1.424)	1.027 (0.727 to 1.425)
Exercise Intervention cycle	-1.115 (-11.6119 to 6.609)	1.225 (0.686 to 3.492)
sample size	-0.684 (-1.816 to 0.275)	0.998 (0.705 to 1.417)
Exercise intervention intensity	0.11 (-0.697 to 0.924)	1.019 (0.733 to 1.395)

Note: *, indicates the presence of a significant impact effect with a significant 95% confidence interval

## Discussion

This systematic review and meta-analysis is the first comprehensive study to examine the effects of different exercise interventions on cravings in individuals with substance use disorders and the associated dose-response relationships. The current review uses a meta-analytic approach to systematically evaluate the effects of different exercise interventions on craving in individuals with substance use disorders. By analyzing the results of a number of randomized controlled trials, each with varying levels of methodological rigor, this study aims to provide an in-depth review of the effectiveness of exercise in reducing cravings.

### Major finding

A meta-analysis of all exercise interventions affecting craving levels was conducted using a frequentist model. The analysis showed that aerobic exercise, when combined with resistance training, as well as high-intensity interval training, had a significant positive effect on craving levels in individuals with substance use disorders. In contrast, traditional Chinese exercise, resistance training alone, and other forms of exercise did not show a significant effect on these craving levels. Furthermore, the data suggested that the combination of aerobic and resistance exercise was more effective than other exercise interventions. In addition, high-intensity interval training appeared to outperform traditional Chinese exercises. Subsequent subgroup analyses examining the differential effects of acute versus long-term aerobic exercise revealed heterogeneity in the results. The results suggested that both long-term and acute aerobic interventions were capable of exerting a significant positive effect on subjects’ drug craving. Subgroup analyses of traditional Chinese exercise interventions further demonstrated that Taijiquan specifically had a significant positive effect on subjects’ craving.

A dose-response model was used to predict the dose-response curve for the total exercise intervention and to derive the effective intervention dose interval. The model estimated the minimum effective dose of the exercise intervention to be 20 METs-min/week, derived from a single aerobic exercise intervention. The maximum effective dose of the exercise intervention was determined to be 320 METs-min/week, and it was observed that once the exercise dose entered the effective interval, the slope of the dose curve began to decrease and the increase in the effect of exercise became extremely slow. Consequently, it was hypothesized that there was a “plateau” in the effect of exercise on the subjects’ desire for the intervention. Second, because the maximum exercise dose used in this study was 320 METs-min/week, it was not possible to further examine the specific effects of the exercise intervention on craving at a higher dose [91].This discrepancy may be due to the suboptimal physical fitness and health status of long-term addicts, which precludes them from benefiting from high-intensity or high-dose exercise interventions. It is imperative that research in this area give due consideration to the safety of subjects [92, 93].

When the dose was analyzed separately for each exercise intervention, the results showed that the dose-response curves for the different types of exercise were remarkably similar. All showed a negative correlation. The minimum effective dose for aerobic exercise was identified as 9.5 METs-min/week, while the optimal dose was found to be 180 METs-min/week. For traditional Chinese exercise, the minimum effective dose was 45 METs-min/week, with an optimal dose of 300 METs-min/week. The combination of aerobic and resistance exercise proved to be the most effective approach, achieving the lowest effective dose of just 20 METs-min/week and an optimal exercise dose of 320 METs-min/week. Additionally, high-intensity interval training recorded a minimum effective dose of 120 METs-min/week. Firstly, the difference in the sample size of the study prevented the dose analysis curves of multiple exercise interventions from presenting as smooth curves, reducing the informativeness of the results. Therefore, in this study, the dose-response curves of aerobic exercise, aerobic combined resistance exercise and Chinese traditional exercise were considered to have the best reference value.

In the final analysis, the present study discovered that the age of the subjects may moderate the intervention effect of exercise to a certain extent. The year of addiction, the percentage of females, the addictive substance, the period of the exercise intervention, the sample size, and the intensity of the exercise intervention did not affect the intervention effect of exercise on craving.

### Possible mechanisms

The impact of exercise on substance craving in individuals with substance use disorders may be attributable to multi-system interaction mechanisms that encompass various levels of neurobiology, metabolic regulation, and psychobehavior.

At the neurobiological level, the results of the epistemic cognition study suggest that improvements in craving in individuals with substance use disorders by acute aerobic exercise may be associated with improvements in attentional network functioning, which showed a significant negative correlation [81, 94]. The rationale for this comes from the fact that neurotransmitter suppression from long-term drug use (e.g., methamphetamine) leads to abnormal changes in an individual’s dopamine system, and that exercise improves conflict networks by activating the brain’s striatum [95, 96]. Secondly, in an animal model, it was observed that exercise led to a reduction in D1 dopamine receptor expression and an increase in D2 receptor expression in the nucleus ambiguus and striatum. Additionally, it was noted that levels of brain-derived neurotrophic factor (BDNF) decreased in mice with a history of cocaine exposure, which in turn reduced the reward salience of drug-associated cues [97–99]. Activation of the glutamatergic system (mGlu5) during exercise upregulates prefrontal cortical transmission to the nucleus ambiguus and may have inhibitory effects on impulsive drug-seeking behavior [100, 101]. Ultimately, the neuroplasticity improved by exercise is particularly important due to the significant correlation between brain-derived neurotrophic factors and the risk of drug addiction [102]. Synthesis and Release of Brain-Derived Neurotrophic Factor (BDNF) Induced During Exercise May Reduce Subjects’ Risk of Substance Addiction [103, 104].

Exercise, in particular, has demonstrated the capacity to enhance mitochondrial function, making it a compelling therapeutic intervention for individuals grappling with substance use disorders. This potential lies in its ability to promote mitochondrial integrity, thereby restoring energy metabolism in the brains of individuals with substance use disorders resulting from chronic substance abuse [105, 106]. Secondly, neuroinflammation in the brain due to substance use disorders is acknowledged as a significant manifestation of neurotoxicity, potentially contributing to cognitive impairment in individuals suffering from these disorders [107, 108]. In animal models, prolonged exercise effectively improved levels of inflammatory factors (e.g., Interleukin-1B, Interleukin-6, and TNF-α) in specific areas of the mouse brain (e.g., cerebral cortex, striatum, and hippocampus) that had been exposed to methamphetamine [29]. A subsequent specific human study found eight weeks of aerobic combined resistance exercise to be effective in improving peripheral inflammation levels in individuals with substance use disorders. However, the findings do not support changes in brain inflammation, and the necessity of future studies to validate these results is paramount [109].

At the psycho-behavioral level, studies have identified a correlation between exercise and improved cravings. This correlation has been attributed, in part, to the efficacy of exercise behavior, which has been demonstrated to promote positive thinking. Meditation, a component of exercise behavior, has also been shown to significantly reduce negative emotions and physiological arousal [78, 110, 111]. This conclusion is further substantiated by the findings of partial functional magnetic resonance imaging evidence, which indicate enhancements in the associated anterior cingulate cortex and striatal brain pathways [112, 113]. Secondly, given that salivary cortisol levels in individuals with substance use disorders have been demonstrated to serve as a reliable predictor of craving levels, a correlation between cortisol levels and cravings was subsequently identified [114]. Exercise has been demonstrated to reduce basal cortisol levels by modulating the HPA axis. This effect may serve to attenuate the stress-induced effects of drug craving [115–117].

## Limitation

1) Certain exercise interventions were addressed in an inadequate number of studies within the existing literature (e.g., high-intensity interval training, aerobic combined with resistance exercise, and resistance exercise alone), which hindered the ability to produce smooth dose-response curves when predicting their effects.

2) While this study examined the potential mechanisms through which exercise influences medication cravings in substance use disorders (SUDs), it was not exhaustive in scope, as the exploration of these mechanisms was not the primary focus. Second, in this paper we neglected to discuss the role of behavioral mechanisms (i.e., distraction theory) in reducing craving, which deserves to be further explored in subsequent research.

3) The current study analyzed the role of exercise intensity as a covariate in the overall intervention effect through regression analysis; however, the results were biased due to inconsistent definitions and delineations of exercise intensity across the included studies. Future investigations should consider utilizing exercise heart rate as a key indicator for defining exercise intensity.

4) The characteristics of the experimental subjects influenced the range of exercise doses, which varied from a minimum of 10 min for a single intervention (typically for special populations) to a maximum of 320 min per week. This variability did not allow for the identification of a desirable “U”-shaped dose-response curve in this study.

## Conclusion

1) The effectiveness of various exercise interventions in reducing craving levels among individuals with substance use disorders shows a dose-response relationship, indicating that total exercise dose correlates with craving levels;

2) Aerobic exercise, aerobic combined with resistance exercise, and high-intensity interval training may serve as effective interventions for improving craving levels in this population, with aerobic combined resistance exercise possibly demonstrating superior effects compared to the other types;

3) For specific populations, all the studies reviewed indicated a lack of larger exercise doses in the interventions employed, underscoring the necessity for future randomized controlled trials to explore and assess the impact of larger doses of exercise interventions.

4) The large or larger-than-large effect sizes identified in this study [aerobic exercise (SMD = -0.73), high-intensity interval exercise (SMD = -2.19), and aerobic combined with resistance exercise (SMD = -1.96)] indicate that the benefits of exercise are highly pronounced at the population level, strongly suggesting significant clinical exploration value. Given that craving is the core mechanism driving relapse, such a significant acute reduction may substantially help patients gain a critical “decision window” when cravings strike, thereby enhancing their ability to resist impulses and prevent relapse.

## Supplementary Information

Below is the link to the electronic supplementary material.

Supplementary Material

## Data Availability

Wang, Chuan (2025), “A Review of the Effect of Different Exercise Types on Medication Craving in Patients with Substance Use Disorders-Based on a Dose-Response Relationship Perspective”, Mendeley Data, V2, 10.17632/bstzgj6yvy.2.

## References

[1] World Drug Report. 2024.

[2] Zhang B, Yan X, Li Y, et al. Trends in methamphetamine use in the Mainland of China, 2006–2015. Front Public Health. 2022;10:852837. 35570894 10.3389/fpubh.2022.852837 PMC9096246

[3] Kim B, Yun J, Park B. Methamphetamine-Induced neuronal damage: neurotoxicity and neuroinflammation. Biomol Ther (Seoul). 2020;28:381–8. 32668144 10.4062/biomolther.2020.044 PMC7457172

[4] Volkow ND, Michaelides M, Baler R. The neuroscience of drug reward and addiction. Physiol Rev. 2019;99:2115–40. 31507244 10.1152/physrev.00014.2018 PMC6890985

[5] Goldstein RZ, Volkow ND. Drug addiction and its underlying Neurobiological basis: neuroimaging evidence for the involvement of the frontal cortex. Am J Psychiatry. 2002;159:1642–52. 12359667 10.1176/appi.ajp.159.10.1642 PMC1201373

[6] Baldo BA, Rose MA. Mechanisms of opioid-induced respiratory depression. Arch Toxicol. 2022;96:2247–60. 35471232 10.1007/s00204-022-03300-7

[7] Kevil CG, Goeders NE, Woolard MD, et al. Methamphetamine use and cardiovascular disease. Arterioscler Thromb Vasc Biol. 2019;39:1739–46. 31433698 10.1161/ATVBAHA.119.312461 PMC6709697

[8] Mladěnka P, Applová L, Patočka J, et al. Comprehensive review of cardiovascular toxicity of drugs and related agents. Med Res Rev. 2018;38:1332–403. 29315692 10.1002/med.21476 PMC6033155

[9] Li J, Jiang W, Zhu R, et al. Depression in Chinese men with methamphetamine dependence: Prevalence, correlates and relationship with alexithymia. J Affect Disord. 2022;319:235–43. 36162653 10.1016/j.jad.2022.09.064

[10] Saffari M, Chang K-C, Chen J-S, et al. Temporal associations between depressive features and self-stigma in people with substance use disorders related to heroin, amphetamine, and alcohol use: a cross-lagged analysis. BMC Psychiatry. 2022;22:815. 36544132 10.1186/s12888-022-04468-z PMC9768939

[11] Schlüter B-S, Alburez-Gutierrez D, Bibbins-Domingo K, et al. Youth experiencing parental death due to drug poisoning and firearm violence in the US, 1999–2020. JAMA. 2024;331:1741–7. 38703404 10.1001/jama.2024.8391 PMC11070062

[12] Lee D, Delcher C, Maldonado-Molina MM, et al. Manners of death in Drug-Related fatalities in Florida. J Forensic Sci. 2016;61:735–42. 27122413 10.1111/1556-4029.12999

[13] Lee NK, Jenner L, Harney A, et al. Pharmacotherapy for amphetamine dependence: A systematic review. Drug Alcohol Depend. 2018;191:309–37. 30173086 10.1016/j.drugalcdep.2018.06.038

[14] Chan B, Freeman M, Kondo K, et al. Pharmacotherapy for methamphetamine/amphetamine use disorder-a systematic review and meta-analysis. Addiction. 2019;114:2122–36. 31328345 10.1111/add.14755

[15] Siefried KJ, Acheson LS, Lintzeris N, et al. Pharmacological treatment of Methamphetamine/Amphetamine dependence: A systematic review. CNS Drugs. 2020;34:337–65. 32185696 10.1007/s40263-020-00711-x PMC7125061

[16] Kampman KM. The treatment of cocaine use disorder. Sci Adv. 2019;5:eaax1532. 31663022 10.1126/sciadv.aax1532 PMC6795516

[17] Koob GF, Le Moal M. Addiction and the brain antireward system. Annu Rev Psychol. 2008;59:29–53. 18154498 10.1146/annurev.psych.59.103006.093548

[18] Koob GF, Volkow ND. Neurobiology of addiction: a neurocircuitry analysis. Lancet Psychiatry. 2016;3:760–73. 27475769 10.1016/S2215-0366(16)00104-8 PMC6135092

[19] Morais APD, Pita IR, Fontes-Ribeiro CA, et al. The Neurobiological mechanisms of physical exercise in methamphetamine addiction. CNS Neurosci Ther. 2018;24:85–97. 29266758 10.1111/cns.12788 PMC6489779

[20] Thompson AB, Stolyarova A, Ying Z, et al. Methamphetamine blocks exercise effects on Bdnf and Drd2 gene expression in frontal cortex and striatum. Neuropharmacology. 2015;99:658–64. 26334786 10.1016/j.neuropharm.2015.08.045 PMC5352165

[21] Curtin D, Taylor EM, Bellgrove MA, et al. Dopamine D2 receptor modulates exercise related effect on cortical Excitation/Inhibition and motor skill acquisition. J Neurosci. 2024;44:e2028232024. 38553046 10.1523/JNEUROSCI.2028-23.2024 PMC11079968

[22] Zlebnik NE, Anker JJ, Carroll ME. Exercise to reduce the escalation of cocaine self-administration in adolescent and adult rats. Psychopharmacology. 2012;224:387–400. 22752381 10.1007/s00213-012-2760-7 PMC3773508

[23] Smith MA, Walker KL, Cole KT, et al. The effects of aerobic exercise on cocaine self-administration in male and female rats. Psychopharmacology. 2011;218:357–69. 21567123 10.1007/s00213-011-2321-5 PMC3752981

[24] Smith MA, Fronk GE, Abel JM, et al. Resistance exercise decreases heroin self-administration and alters gene expression in the nucleus accumbens of heroin-exposed rats. Psychopharmacology. 2018;235:1245–55. 29396617 10.1007/s00213-018-4840-9 PMC5871570

[25] Qi L, Tian Z-H, Yue Y, et al. Effects of acute exercise on craving and cortical hemodynamics under drug-cue exposure in MA-dependent individuals. Neurosci Lett. 2022;781:136672. 35504405 10.1016/j.neulet.2022.136672

[26] Haberstroh C, Weider S, Flemmen G, et al. The effect of high-intensity interval training on cognitive function in patients with substance use disorder: study protocol for a two-armed randomized controlled trial. Front Sports Act Living. 2022;4:954561. 36570498 10.3389/fspor.2022.954561 PMC9780390

[27] Rung JM, Peck S, Hinnenkamp J, et al. Changing delay discounting and impulsive choice: implications for Addictions, Prevention, and human health. Perspect Behav Sci. 2019;42:397–417. 31650104 10.1007/s40614-019-00200-7 PMC6768935

[28] Gray JC, MacKillop J. Impulsive delayed reward discounting as a genetically-influenced target for drug abuse prevention: a critical evaluation. Front Psychol. 2015;6:1104. 26388788 10.3389/fpsyg.2015.01104 PMC4554956

[29] Li Y, Re G-F, Zhao Y, et al. Long-term exercise at different intensities can reduce the inflammatory response in the brains of methamphetamine-treated mice. Biochem Biophys Res Commun. 2022;613:201–6. 35598376 10.1016/j.bbrc.2022.05.042

[30] Richards JR. Cannabinoid hyperemesis syndrome: A disorder of the HPA axis and sympathetic nervous system? Med Hypotheses. 2017;103:90–5. 28571820 10.1016/j.mehy.2017.04.018

[31] Athanasiou N, Bogdanis GC, Mastorakos G. Endocrine responses of the stress system to different types of exercise. Rev Endocr Metab Disord. 2023;24:251–66. 36242699 10.1007/s11154-022-09758-1 PMC10023776

[32] Smelson D, Chen KW, Ziedonis D, et al. A pilot study of qigong for reducing cocaine craving early in recovery. J Altern Complement Med. 2013;19:97–101. 22757968 10.1089/acm.2012.0052 PMC3576894

[33] Wang D, Zhou C, Chang Y-K. Acute exercise ameliorates craving and inhibitory deficits in methamphetamine: an ERP study. Physiol Behav. 2015;147:38–46. 25846839 10.1016/j.physbeh.2015.04.008

[34] Wang D, Zhou C, Zhao M, et al. Dose-response relationships between exercise intensity, cravings, and inhibitory control in methamphetamine dependence: an erps study. Drug Alcohol Depend. 2016;161:331–9. 26946990 10.1016/j.drugalcdep.2016.02.023

[35] Chen Y, Liu T, Zhou C. Effects of 12-week aerobic exercise on cue-induced drug craving in methamphetamine-dependent patients and the moderation effect of working memory. Ment Health Phys Act. 2021;21:100420.

[36] Jia D, Xu Y. Effects of an 8-week Baduanjin intervention combined with low-carbohydrates diet among overweight people who struggle with drug addiction. Front Public Health. 2022;10:989519. 36339240 10.3389/fpubh.2022.989519 PMC9633992

[37] Page MJ, McKenzie JE, Bossuyt PM, et al. The PRISMA 2020 statement: an updated guideline for reporting systematic reviews. BMJ. 2021:n71. 10.1136/bmj.n71 PMC8005924 33782057

[38] Ardern CL, Büttner F, Andrade R, et al. Implementing the 27 PRISMA 2020 statement items for systematic reviews in the sport and exercise medicine, musculoskeletal rehabilitation and sports science fields: the persist (implementing Prisma in Exercise, Rehabilitation, sport medicine and sports science) guidance. Br J Sports Med. 2022;56:175–95. 34625401 10.1136/bjsports-2021-103987 PMC8862073

[39] Cumpston M, Li T, Page MJ, et al. Updated guidance for trusted systematic reviews: a new edition of the Cochrane handbook for systematic reviews of interventions. Cochrane Database Syst Rev. 2019;10:ED000142. 31643080 10.1002/14651858.ED000142 PMC10284251

[40] Shi J, Luo D, Wan X, et al. Detecting the skewness of data from the five-number summary and its application in meta-analysis. Stat Methods Med Res. 2023;32:1338–60. 37161735 10.1177/09622802231172043

[41] Leal-Martín J, Muñoz-Muñoz M, Keadle SK, et al. Resting oxygen uptake value of 1 metabolic equivalent of task in older adults: A systematic review and descriptive analysis. Sports Med. 2022;52:331–48. 34417980 10.1007/s40279-021-01539-1

[42] Matthews CE, Moore SC, Arem H, et al. Amount and intensity of Leisure-Time physical activity and lower cancer risk. J Clin Oncol. 2020;38:686–97. 31877085 10.1200/JCO.19.02407 PMC7048166

[43] Ainsworth BE, Haskell WL, Herrmann SD, et al. 2011 compendium of physical activities: a second update of codes and MET values. Med Sci Sports Exerc. 2011;43:1575–81. 21681120 10.1249/MSS.0b013e31821ece12

[44] Jeong S-W, Kim S-H, Kang S-H, et al. Mortality reduction with physical activity in patients with and without cardiovascular disease. Eur Heart J. 2019;40:3547–55. 31504416 10.1093/eurheartj/ehz564 PMC6855138

[45] Higgins JPT, Jackson D, Barrett JK, et al. Consistency and inconsistency in network meta-analysis: concepts and models for multi-arm studies. Res Synth Methods. 2012;3:98–110. 26062084 10.1002/jrsm.1044 PMC4433772

[46] Fudala PJ, Woody GW. Recent advances in the treatment of opiate addiction. Curr Psychiatry Rep. 2004;6:339–46. 15355756 10.1007/s11920-004-0020-1

[47] Sterne JAC, Savović J, Page MJ, et al. RoB 2: a revised tool for assessing risk of bias in randomised trials. BMJ. 2019;366:l4898. 31462531 10.1136/bmj.l4898

[48] Higgins JP, Del Giovane C, Chaimani A, et al. Evaluating the quality of evidence from a network Meta-Analysis. Value Health. 2014;17:A324. 27200533 10.1016/j.jval.2014.08.572

[49] Guyatt GH, Oxman AD, Vist GE, et al. GRADE: an emerging consensus on rating quality of evidence and strength of recommendations. BMJ. 2008;336:924–6. 18436948 10.1136/bmj.39489.470347.AD PMC2335261

[50] Higgins JPT, Thompson SG. Quantifying heterogeneity in a meta-analysis. Stat Med. 2002;21:1539–58. 12111919 10.1002/sim.1186

[51] Lin L, Chu H, Murad MH, et al. Empirical comparison of publication bias tests in Meta-Analysis. J Gen Intern Med. 2018;33:1260–7. 29663281 10.1007/s11606-018-4425-7 PMC6082203

[52] Owen RK, Bradbury N, Xin Y, et al. MetaInsight: an interactive web-based tool for analyzing, interrogating, and visualizing network meta‐analyses using R‐shiny and Netmeta. Res Synthesis Methods. 2019;10:569–81. 10.1002/jrsm.1373 PMC6973101 31349391

[53] Salanti G, Ades AE, Ioannidis JPA. Graphical methods and numerical summaries for presenting results from multiple-treatment meta-analysis: an overview and tutorial. J Clin Epidemiol. 2011;64:163–71. 20688472 10.1016/j.jclinepi.2010.03.016

[54] Mawdsley D, Bennetts M, Dias S, et al. Model-Based network Meta-Analysis: A framework for evidence synthesis of clinical trial data. CPT Pharmacometrics Syst Pharmacol. 2016;5:393–401. 27479782 10.1002/psp4.12091 PMC4999602

[55] Wheeler DC, Hickson DA, Waller LA. Assessing local model adequacy in bayesian hierarchical models using the partitioned deviance information criterion. Comput Stat Data Anal. 2010;54:1657–71. 21243121 10.1016/j.csda.2010.01.025 PMC3020089

[56] Pedder H, Dias S, Bennetts M, et al. Modelling time-course relationships with multiple treatments: Model-based network meta-analysis for continuous summary outcomes. Res Synth Methods. 2019;10:267–86. 31013000 10.1002/jrsm.1351 PMC6563489

[57] Borg DN, Impellizzeri FM, Borg SJ, et al. Meta-analysis prediction intervals are under reported in sport and exercise medicine. Scand J Med Sci Sports. 2024;34:e14603. 38501202 10.1111/sms.14603

[58] Donegan S, Dias S, Tudur-Smith C, et al. Graphs of study contributions and covariate distributions for network meta‐regression. Res Synthesis Methods. 2018;9:243–60. 10.1002/jrsm.1292 PMC6001528 29377598

[59] Nevill CR, Cooper NJ, Sutton AJ. A multifaceted graphical display, including treatment ranking, was developed to aid interpretation of network meta-analysis. J Clin Epidemiol. 2023;157:83–91. 36870376 10.1016/j.jclinepi.2023.02.016

[60] Sung Y-T, Wu J-S. The visual analogue scale for Rating, ranking and Paired-Comparison (VAS-RRP): A new technique for psychological measurement. Behav Res Methods. 2018;50:1694–715. 29667082 10.3758/s13428-018-1041-8 PMC6096654

[61] De La Garza R, Yoon JH, Thompson-Lake DGY, et al. Treadmill exercise improves fitness and reduces craving and use of cocaine in individuals with concurrent cocaine and tobacco-use disorder. Psychiatry Res. 2016;245:133–40. 27541349 10.1016/j.psychres.2016.08.003 PMC5067203

[62] Wang D, Zhu T, Zhou C, et al. Aerobic exercise training ameliorates craving and inhibitory control in methamphetamine dependencies: A randomized controlled trial and event-related potential study. Psychol Sport Exerc. 2017;30:82–90.

[63] Wang D, Zhu T. Effects of aerobic exercise on physical fitness, craving, and emotional state in methamphetamine-dependent individuals. Sports Sci. 2017;37(7):50–9. 10.16469/j.css.201707007.

[64] Ellingsen MM, Johannesen SL, Martinsen EW, et al. Effects of acute exercise on drug craving, self-esteem, mood and affect in adults with poly‐substance dependence: feasibility and preliminary findings. Drug Alcohol Rev. 2018;37:789–93. 29869351 10.1111/dar.12818

[65] Lu C, Dong W, Zheng L, et al. Effects of exercise intervention to activate immune stress and dopamine levels on the mental health of compulsorily isolated drug addicts with amphetamine addiction. Chin J Sports Med. 2019;38(9):762–70. 10.16038/j.1000-6710.2019.09.005.

[66] Gong D, Qin L, Zhu T, et al. Effects of short-duration aerobic exercise on craving, emotional state and neurotransmitters in methamphetamine-dependent individuals. China Sports Sci Technol. 2019;55(05):56–64. 10.16470/j.csst.2019885.

[67] Wang D, Zhu T, Chen J, et al. Acute aerobic exercise ameliorates cravings and inhibitory control in heroin addicts: evidence from Event-Related potentials and frequency bands. Front Psychol. 2020;11:561590. 33101132 10.3389/fpsyg.2020.561590 PMC7554636

[68] Ellingsen MM, Clausen T, Johannesen SL, et al. Effects of acute exercise on drug craving in adults with poly-substance use disorder. A randomized controlled trial. Ment Health Phys Act. 2021;21:100423.

[69] Xu X, Ding X, Chen L, et al. The transcranial direct current stimulation over prefrontal cortex combined with the cognitive training reduced the cue-induced craving in female individuals with methamphetamine use disorder: A randomized controlled trial. J Psychiatr Res. 2021;134:102–10. 33383492 10.1016/j.jpsychires.2020.12.056

[70] Zhou YU, Finlayson G, Liu X, et al. Effects of acute dance and aerobic exercise on drug craving and food reward in women with methamphetamine dependence. Med Sci Sports Exerc. 2021;53:2245–53. 34115731 10.1249/MSS.0000000000002723

[71] Salem BA, Gonzales-Castaneda R, Ang A, et al. Craving among individuals with stimulant use disorder in residential social model-based treatment - Can exercise help? Drug Alcohol Depend. 2022;231:109247. 34999268 10.1016/j.drugalcdep.2021.109247 PMC10978100

[72] Wang M, Chen Y, Xu Y, et al. A randomized controlled trial evaluating the effect of Tai Chi on the drug craving in women. Int J Ment Health Addict. 2022:1–13. 10.1007/s11469-022-00917-8 PMC9469824 36119946

[73] Zhu T, Tao W, Peng B, et al. Effects of a Group-Based aerobic exercise program on the cognitive functions and emotions of substance use disorder patients: a randomized controlled trial. Int J Ment Health Addict. 2022;20:2349–65.

[74] Chen Y, Wang X, Zhou C. Effects of different exercise patterns on drug craving in female methamphetamine-dependent patients: evidence from behavior and fNIRS. Ment Health Phys Act. 2023;25:100534.

[75] Li H, Wang C, Huang X, et al. Chan-Chuang and resistance exercise for drug rehabilitation: a randomized controlled trial among Chinese male methamphetamine users. Front Public Health. 2023;11:1180503. 37965508 10.3389/fpubh.2023.1180503 PMC10642185

[76] Guo J, Zhang L, Zhang L, et al. Effect of interactive exergame training on physical fitness and executive function among men with substance use disorder in rehabilitation center. Ment Health Phys Act. 2024;26:100598.

[77] Wang M, Chen Y, Xu Y, et al. A randomized controlled trial evaluating the effect of Tai Chi on the drug craving in women. Int J Ment Health Addict. 2024;22:1103–15. 10.1007/s11469-022-00917-8 PMC9469824 36119946

[78] Zhang L, Zeng H, Sun Y, et al. Effect of Tai Chi compared to running on drug Cravings, attention Bias, and physical fitness in men with methamphetamine use disorder. Healthc (Basel). 2024;12:1653. 10.3390/healthcare12161653 PMC11353623 39201211

[79] He M, Wang L, Xu D, et al. Long-Term High-Intensity interval training intervention improves emotional conflict control in association with right ventrolateral prefrontal activation in males with methamphetamine use disorder: A randomized controlled trial. Scand J Med Sci Sports. 2024;34:e70006. 39707624 10.1111/sms.70006

[80] Liu X, Huang P, Wang Y, et al. Effects of aerobic exercise combined with attentional bias modification in the care of male patients with a methamphetamine use disorder. UJAN. 2024;35:E2–14. 10.1097/JAN.0000000000000565 38574107

[81] Li M, Jin J, Zhai X, et al. Acute aerobic exercise ameliorates craving and attentional function in individuals with methamphetamine use disorders. Physiol Behav. 2025;290:114775. 39631450 10.1016/j.physbeh.2024.114775

[82] Sussner BD, Smelson DA, Rodrigues S, et al. The validity and reliability of a brief measure of cocaine craving. Drug Alcohol Depend. 2006;83:233–7. 16384655 10.1016/j.drugalcdep.2005.11.022

[83] Malagodi BM, Greguol M, Tavares VDO, et al. Can different types of acute physical exercise at moderate intensity influence the inhibitory control and craving levels in individuals with substance use disorder? J Drug Issues. 2024. 10.1177/00220426241248355.

[84] Voris J, Elder I, Sebastian P. A simple test of cocaine craving and related responses. J Clin Psychol. 1991;47:320–3. 2030141 10.1002/1097-4679(199103)47:2<320::aid-jclp2270470221>3.0.co;2-f

[85] Heishman SJ, Evans RJ, Singleton EG, et al. Reliability and validity of a short form of the marijuana craving questionnaire. Drug Alcohol Depend. 2009;102:35–40. 19217724 10.1016/j.drugalcdep.2008.12.010 PMC2694410

[86] Wilson SD, Collins RL, Prince MA, et al. Effects of exercise on experimentally manipulated craving for cannabis: A preliminary study. Exp Clin Psychopharmacol. 2018;26:456–66. 29792472 10.1037/pha0000200 PMC6162100

[87] James D, Davies G, Willner P. The development and initial validation of a questionnaire to measure craving for amphetamine. Addiction. 2004;99:1181–8. 15317639 10.1111/j.1360-0443.2004.00819.x

[88] Zhang Z, Zhu D. Effect of Taijiquan exercise on rehabilitation of male Amphetamine-Type addicts. Evid Based Complement Alternat Med. 2020;2020:8886562. 33293997 10.1155/2020/8886562 PMC7690993

[89] Brellenthin AG, Crombie KM, Hillard CJ, et al. Psychological and endocannabinoid responses to aerobic exercise in substance use disorder patients. Subst Abus. 2021;42:272–83. 31729933 10.1080/08897077.2019.1680480 PMC7225058

[90] Heinz AJ, Epstein DH, Schroeder JR, et al. Heroin and cocaine craving and use during treatment: measurement validation and potential relationships. J Subst Abuse Treat. 2006;31:355–64. 17084789 10.1016/j.jsat.2006.05.009

[91] Bull FC, Al-Ansari SS, Biddle S, et al. World health organization 2020 guidelines on physical activity and sedentary behaviour. Br J Sports Med. 2020;54:1451–62. 33239350 10.1136/bjsports-2020-102955 PMC7719906

[92] Morris L, Stander J, Ebrahim W, et al. Effect of exercise versus cognitive behavioural therapy or no intervention on anxiety, depression, fitness and quality of life in adults with previous methamphetamine dependency: a systematic review. Addict Sci Clin Pract. 2018;13:4. 29338767 10.1186/s13722-018-0106-4 PMC5771022

[93] Huang J, Zheng Y, Gao D, et al. Effects of exercise on Depression, Anxiety, cognitive Control, Craving, physical fitness and quality of life in Methamphetamine-Dependent patients. Front Psychiatry. 2019;10:999. 32047445 10.3389/fpsyt.2019.00999 PMC6997340

[94] Meng F, Xie C, Qiu F, et al. Effects of physical activity level on attentional networks in young adults. Int J Environ Res Public Health. 2022;19:5374. 35564768 10.3390/ijerph19095374 PMC9105944

[95] Balleine BW, Delgado MR, Hikosaka O. The role of the dorsal striatum in reward and decision-making. J Neurosci. 2007;27:8161–5. 17670959 10.1523/JNEUROSCI.1554-07.2007 PMC6673072

[96] Volkow ND, Fowler JS, Wang GJ, et al. Imaging dopamine’s role in drug abuse and addiction. Neuropharmacology. 2009;56(Suppl 1):3–8. 18617195 10.1016/j.neuropharm.2008.05.022 PMC2696819

[97] Robison LS, Swenson S, Hamilton J, et al. Exercise reduces dopamine D1R and increases D2R in rats: implications for addiction. Med Sci Sports Exerc. 2018;50:1596–602. 29613999 10.1249/MSS.0000000000001627

[98] Tyler J, Podaras M, Richardson B, et al. High intensity interval training exercise increases dopamine D2 levels and modulates brain dopamine signaling. Front Public Health. 2023;11:1257629. 38192549 10.3389/fpubh.2023.1257629 PMC10773799

[99] Strickland JC, Abel JM, Lacy RT, et al. The effects of resistance exercise on cocaine self-administration, muscle hypertrophy, and BDNF expression in the nucleus accumbens. Drug Alcohol Depend. 2016;163:186–94. 27137405 10.1016/j.drugalcdep.2016.04.019 PMC4880539

[100] Abel JM, Nesil T, Bakhti-Suroosh A, et al. Mechanisms underlying the efficacy of exercise as an intervention for cocaine relapse: a focus on mGlu5 in the dorsal medial prefrontal cortex. Psychopharmacology. 2019;236:2155–71. 31161451 10.1007/s00213-019-05208-0 PMC6626681

[101] Pomierny-Chamiolo L, Miszkiel J, Frankowska M, et al. Cocaine self-administration, extinction training and drug-induced relapse change metabotropic glutamate mGlu5 receptors expression: evidence from radioligand binding and immunohistochemistry assays. Brain Res. 2017;1655:66–76. 27871824 10.1016/j.brainres.2016.11.014

[102] Cotman CW, Berchtold NC. Exercise: a behavioral intervention to enhance brain health and plasticity. Trends Neurosci. 2002;25:295–301. 12086747 10.1016/s0166-2236(02)02143-4

[103] Lynch WJ, Peterson AB, Sanchez V, et al. Exercise as a novel treatment for drug addiction: a Neurobiological and stage-dependent hypothesis. Neurosci Biobehav Rev. 2013;37:1622–44. 23806439 10.1016/j.neubiorev.2013.06.011 PMC3788047

[104] Xiao Y, Zhu Y, Li Y. Elevation of DNA methylation in the promoter regions of the Brain-Derived neurotrophic factor gene is associated with heroin addiction. J Mol Neurosci. 2021;71:1752–60. 34173192 10.1007/s12031-021-01864-0

[105] Thornton C, Grad E, Yaka R. The role of mitochondria in cocaine addiction. Biochem J. 2021;478:749–64. 33626141 10.1042/BCJ20200615

[106] Memme JM, Erlich AT, Phukan G, et al. Exercise and mitochondrial health. J Physiol. 2021;599:803–17. 31674658 10.1113/JP278853

[107] Jayanthi S, Daiwile AP, Cadet JL. Neurotoxicity of methamphetamine: main effects and mechanisms. Exp Neurol. 2021;344:113795. 34186102 10.1016/j.expneurol.2021.113795 PMC8338805

[108] Rodrigues LCM, Gobira PH, de Oliveira AC, et al. Neuroinflammation as a possible link between cannabinoids and addiction. Acta Neuropsychiatr. 2014;26:334–46. 25455257 10.1017/neu.2014.24

[109] Wang J, Lu C, Zheng L, et al. Peripheral inflammatory biomarkers of methamphetamine withdrawal patients based on the Neuro-Inflammation hypothesis: the possible improvement effect of exercise. Front Psychiatry. 2021;12:795073. 35002809 10.3389/fpsyt.2021.795073 PMC8733583

[110] Taylor-Piliae RE, Finley BA. Tai Chi exercise for psychological well-being among adults with cardiovascular disease: A systematic review and meta-analysis. Eur J Cardiovasc Nurs. 2020;19:580–91. 32515204 10.1177/1474515120926068

[111] Priddy SE, Howard MO, Hanley AW, et al. Mindfulness meditation in the treatment of substance use disorders and preventing future relapse: neurocognitive mechanisms and clinical implications. Subst Abuse Rehabil. 2018;9:103–14. 30532612 10.2147/SAR.S145201 PMC6247953

[112] Lorenzetti V, Gaillard A, Beyer E, et al. Do mindfulness-based interventions change brain function in people with substance dependence? A systematic review of the fMRI evidence. BMC Psychiatry. 2023;23:407. 37286936 10.1186/s12888-023-04789-7 PMC10246321

[113] Murphy E, Poudel G, Ganesan S, et al. Real-time fMRI-based neurofeedback to restore brain function in substance use disorders: A systematic review of the literature. Neurosci Biobehav Rev. 2024;165:105865. 39197715 10.1016/j.neubiorev.2024.105865

[114] Sampedro-Piquero P, Vicario S, Pérez-Rivas A, et al. Salivary cortisol levels are associated with craving and cognitive performance in Cocaine-Abstinent subjects: A pilot study. Brain Sci. 2020;10:682. 32992573 10.3390/brainsci10100682 PMC7600918

[115] Walter M, Bentz D, Schicktanz N, et al. Effects of cortisol administration on craving in heroin addicts. Transl Psychiatry. 2015;5:e610. 26218852 10.1038/tp.2015.101 PMC5068724

[116] Moyers SA, Hagger MS. Physical activity and cortisol regulation: A meta-analysis. Biol Psychol. 2023;179:108548. 37001634 10.1016/j.biopsycho.2023.108548

[117] Wang J, Zou Z. Establishment of a biomarker of peripheral stress in opioid addicts based on the hypothalamic-pituitary-adrenal axis-The improvement effect of exercise. Front Psychiatry. 2022;13:1072896. 36569629 10.3389/fpsyt.2022.1072896 PMC9768425

