# Supplementary material for: Effects of different exercise types on craving in substance use disorder patients with drug dependence -network meta-analysis and dose-response relationships based on frequentist and Bayesian models
Source: Addict Sci Clin Pract. 2025 Dec 18;21:12. doi: 10.1186/s13722-025-00639-x (PMC12825257; doi:10.1186/s13722-025-00639-x)
Supplement: Supplementary file 1 — Supplementary Material [file 13722_2025_639_MOESM1_ESM.docx]

**Supplementary material**

catalogs

[1. Study characteristics and meta-analysis results 1](#_Toc3957)

[2 .Dose-response analysis of exercise interventions 6](#_Toc15054)

[2.1 Examining network Connectivity 7](#_Toc15940)

[2.2 Examining the dose-response relationship 7](#_Toc8400)

[2.2.1 Total Exercise Dose Relationship 7](#_Toc31250)

[2.2.2 Individual Motion Measurement Relationships 8](#_Toc21642)

[3. Late estimates 9](#_Toc6036)

[3.1 Deviation maps 9](#_Toc1938)

[3.1.1 Total Motion Deviation Chart 9](#_Toc15092)

[3.1.2 Deviation charts for individual sports 9](#_Toc19115)

[3.2 Fitting value monitoring 10](#_Toc16931)

[3.2.1 Monitoring of total motion fit values 10](#_Toc15185)

[3.2.2 Monitoring of the fitted values for each motion 10](#_Toc30389)

[3.3 Ranking 11](#_Toc16046)

[3.3.1 Ranking of the emax model for each exercise intervention 11](#_Toc27529)

[3.3.2 Ranking of the ed50 model for each exercise intervention 11](#_Toc15636)

[3.3.3 Ranking curves based on two models 12](#_Toc19663)

[4. Prediction 12](#_Toc10870)

[4.1 Predictive effect curves 13](#_Toc23854)

[4.1.1 Prediction curves for total exercise dose 13](#_Toc19314)

[4.1.2 Prediction curves for the effects of each exercise intervention 13](#_Toc28943)

[4.2 Predictive effect curves with specific effect intervals 14](#_Toc25581)

[5. Ranking predicted response 15](#_Toc352)

[5.1 Overall projections 15](#_Toc9043)

[5.2 Projections 15](#_Toc31047)

[6. Consistency Testing 16](#_Toc1006)

[7. Reporting bias and GRADE grade quality 17](#_Toc16034)

[7.1 Risk of bias 17](#_Toc15867)

[7.2 GRADE summary of included studies 19](#_Toc13124)

[8 Publication Bias 20](#_Toc13411)

[8.1 Aerobic exercise publication bias 20](#_Toc7495)

[8.2 Traditional campaign publication bias 20](#_Toc12356)

[9. Search example(Pubmed) 21](#_Toc5887)

1. **Study characteristics and meta-analysis results**

| Table 1: Study Characteristics | | | | |
| --- | --- | --- | --- | --- |
| Author(years) | Subject characteristics | addictive substance | Exercise intervention | Outcomes |
| Smelson(2013) | Cocaine-dependent adults, N=84 (M=81, F=3), Age: 27-49 | cocaine (loanword) | Extracorporeal qigong therapy, 2 weeks | CCQ, VCCS, BDI, STAI |
| Wang(2015) | Methamphetamine-dependent adults, N=24, (M=20, F=4), Age: 25-37 | methamphetamine | Moderate intensity aerobic exercise (immediate and 50min) | VAS |
| Wang(2016) | Methamphetamine-dependent adults, N=92 (M=78, F=14), Age: 18-40 | methamphetamine | Low, moderate and high intensity aerobic exercise (immediate and 50min) | VAS, RPE |
| De La Garza (2016) | Cocaine-dependent adults, N=24 (M=19, F=5), Age: 35-52 | cocaine | Aerobics, 4 weeks | VAS, HR, |
| Wang(2017)A | Methamphetamine dependent adults, N=50 (M=44, F=6), Age: 24- 45 | methamphetamine | Aerobic exercise, 12 weeks | VAS, HAMA, BDI |
| Wang(2017)B | Methamphetamine-dependent adults, N=50 (M=44, F=6), Age: 23- 43 | methamphetamine | Aerobic exercise, 12 weeks | VAS, Inhibitory Control Measures, EEG, ERP |
| Ellingsen(2018) | Adults with substance use disorders, N=9 (M=9, F=0), Age: 31-44 | multi-drug | Aerobic exercise (immediate and 40min) | VAS, RPE |
| Wilson(2018) | Marijuana-dependent adults, N=46 (M=35, F=11), Age: 18-25 | hemp | Low, moderate and high intensity aerobic exercise (30min) | MCQ, |
| Lu(2019) | Amphetamine-dependent adults, N=92 (M=92,F=0), Age: 30-32 | amphetamine (medical) | Moderate-intensity aerobic, resistance and flexibility exercise, 12 weeks | VAS, SDS, SAS, SCL-90 |
| Gong(2019) | Methamphetamine dependent adults, N=26, Age: 25-42 | methamphetamine | Aerobics, instant | VAS, POMS |
| Wang(2020) | Heroin-dependent adults, N=60 (M=60, F=0), Age: 30-32 | heroin (loanword) | Aerobic exercise, immediate and 60 min. | VAS, the Go/No-Go task |
| Zhang(2020) | Amphetamine-dependent adults, N=72 (M=72, F=0), Age: 21-60 | amphetamine (medical) | Taijiquan | DSQ, BDI, SAS, grip strength，standing on one feet，body flexion，vital capacity |
| Brellenthin(2021) | Substance Use Disorders (SUDs), N=21 (M=12, F=9), Age: 25-47 | Tobacco, alcohol, marijuana, cocaine, ecstasy, etc. | Moderate-intensity aerobic exercise, 6 weeks | Craving questionnaires-short forms, PHQ, GAD-7 |
| Chen(2021) | Methamphetamine-dependent adults, N=57 (M=52, F=0), Age: 20-45 | methamphetamine | Aerobic exercise, 12 weeks | VAS, the Go/No-Go task, Working memory, Stroop task, 2-back task |
| Ellingsen(2021) | Adults with substance use disorders, N=36 (M=30, F=6), Age: 29-45 | multi-drug | Soccer and circuit practice (immediate and after 4 hours) | VAS, RPE, Mood |
| Xu(2021) | Methamphetamine-dependent adults, N=73 (M=0, F=73), Age: 25-42 | methamphetamine | Aerobic exercise, 4 weeks | VAS, Cognitive Function |
| Zhou(2021) | Methamphetamine-dependent adults, N=37 (M=0, F=37), Age: 20-32 | methamphetamine | Moderate-intensity dance and aerobic exercise, instantly | VAS |
| Salem(2022) | Methamphetamine-dependent adults, N=135 (M=96, F=39), Age: 24-38 | methamphetamine | Aerobic and resistance exercise, 8 weeks | VAS, Depression |
| Wang(2022) | Adults with substance-based mental disorders, N=95 (M=0, F=95), Age: 24-38 | methamphetamine | TaiJiquan, 12 weeks | VAS |
| Zhu(2022) | Adults with Substance Use Disorders, N=77 (M=77, F=0), Age: 28-43 | methamphetamine | Aerobic exercise, 12 weeks | VAS, BDI, Hamilton Anxiety Rating Scale, Physical fitness |
| Jia(2022) | Substance Abuse Obese or Overweight Adults, N=54 (M=54, F=0), Age: 31-40 | multi-drug | Baduanjin, 8 weeks | VAS |
| Chen(2023) | Methamphetamine-dependent adults, N=40 (M=0, F=40), Age: 20-32 | methamphetamine | Aerobics and moderate-intensity power cycling, instantly | VAS, fNIRS |
| Li(2023) | Methamphetamine-dependent adults, N=76 (M=76, F=0), Age: 20-32 | methamphetamine | Chan-Chuang and resistance exercise, 8 weeks | VAS, mental wellbeing, sleep quality, HR, SBP, DBP, MAP,BMI, Vital capacity, Grip Strength, Balance, Vertical jump. |
| Guo(2024) | Adults with substance use disorders, N=44 (M=44, F=0), Age: 27-55 | multi-drug | Moderate Intensity Exercise Games, 12 weeks | VAS, Execution function, Physical fitness. |
| Malagodi(2024) | Adults with substance use disorders, N=43 (M=32, F=11), Age: 18-35 | multi-drug | Aerobic and Functional Exercise, Instantly | CCQ, the Go/No-Go task |
| Wang(2024) | Adults with substance use disorders, N=95 (M=0, F=95), Age: 24-43 | multi-drug | TaiJiquan, 12 weeks | VAS |
| Zhang(2024) | Methamphetamine-dependent adult, N=44 (M=44, F=0), Age: 29-50 | methamphetamine | TaiJiquan, 12 weeks | VAS, attention bias, Physical fitness |
| He(2024) | Methamphetamine-dependent adults, N=40 (M=40, F=0), Age: 25-37 | methamphetamine | HIIT, 36 weeks | VAS |
| Liu(2024) | Methamphetamine-dependent adults, N=89 (M=89, F=0), Age: 24-41 | methamphetamine | Aerobic exercise, 8 weeks | VAS, Cognitive function,The Self-Rating Depression Scale, The Self-Rating Anxiety Scale |
| Li(2025) | Methamphetamine-dependent adults, N=32 (M=32, F=0), Age: 24-41 | methamphetamine | Aerobics, instant | VAS, HR, RPE, ANT |
| Note: CCQ: Cocaine Craving Questionnaire Brief. MCQ: Marijuana Craving Questionnaire – Short Form. HAMA:Hamilton Anxiety Scale. VCSS: Voris Cocaine Craving Scale. STAI: Spielberger State-Trait Anxiety Inventory-State only. BDI: Beck Depression Inventory. PHQ: Patient Health Questionnaire. GAD-7: Generalized Anxiety Disorder Survey. POMS: profile of mood states | | | | |

**Figure 1: Overall effect of exercise intervention on drug craving in subjects**

**
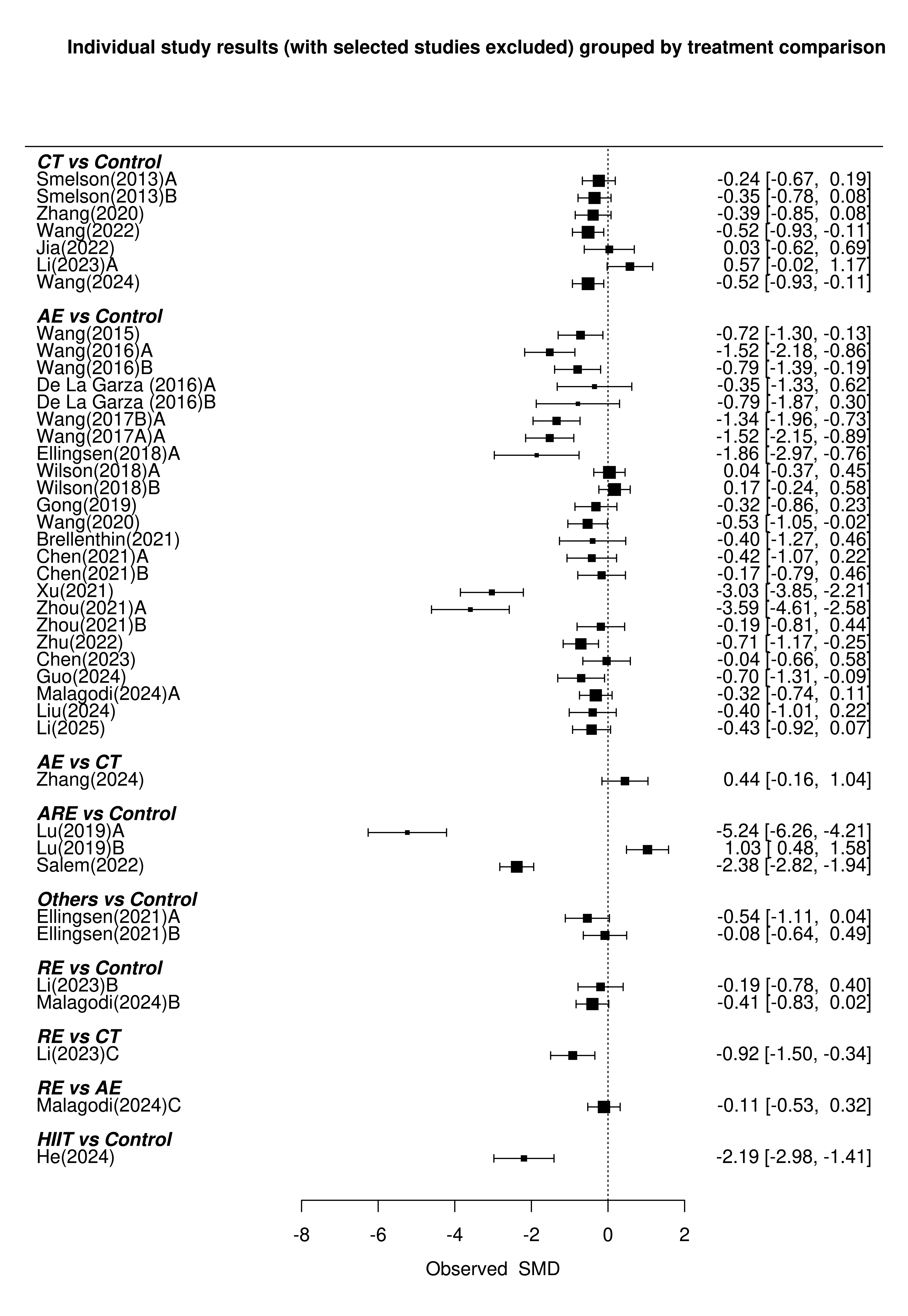
**

**Figure 2: Effect of Traditional Exercise Intervention on Subjects' Drug Cravings**

**
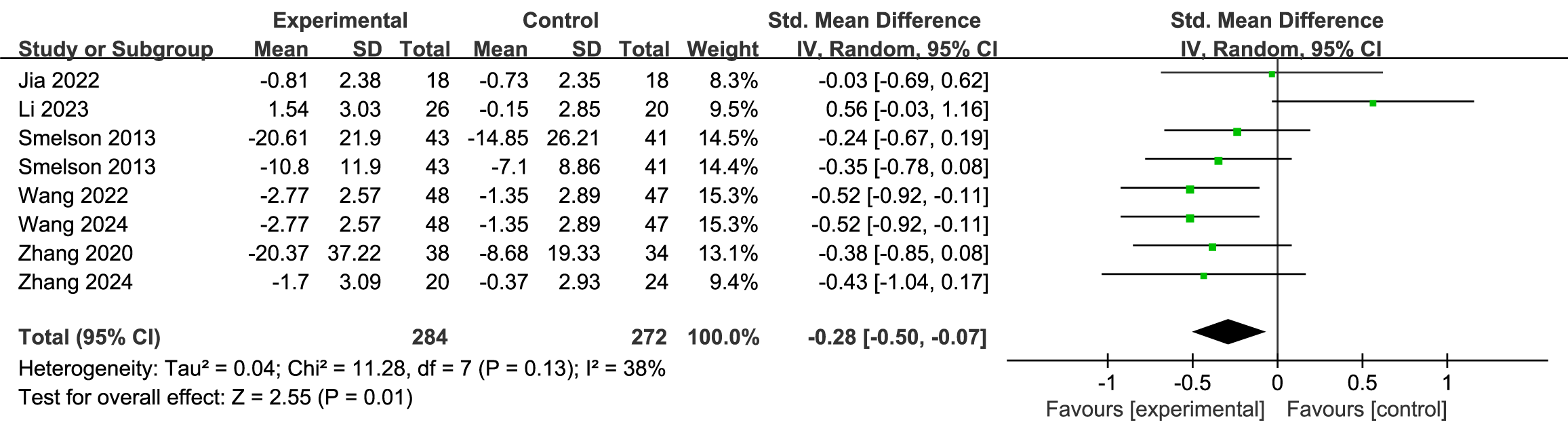
**

**Figure 3: Effect of Aerobic Exercise Intervention on Subjects' Drug Cravings**

**
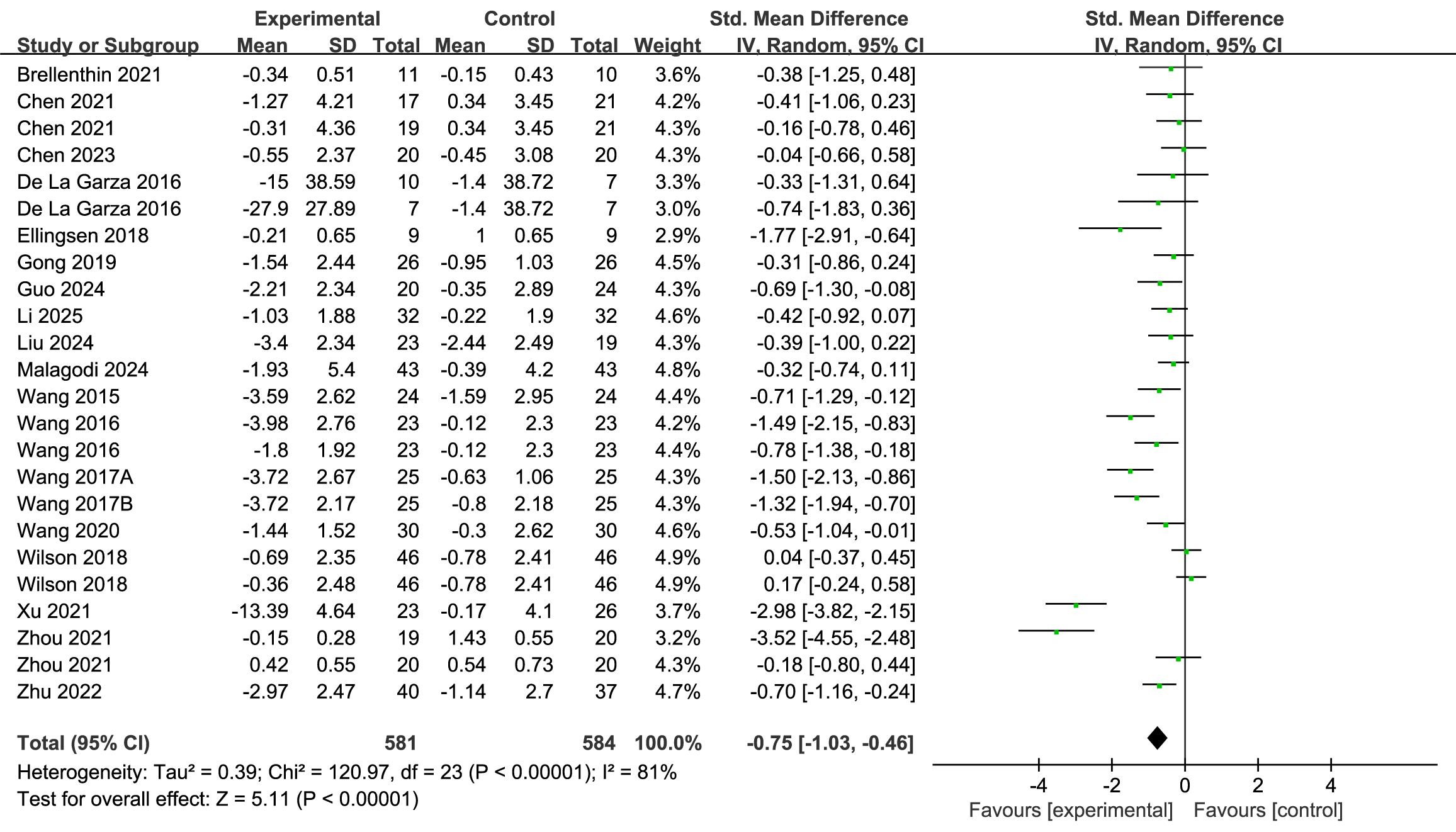
**

**Figure 4: Sensitivity analysis of the effect of aerobic exercise on the effect of drug craving in subjects**

**
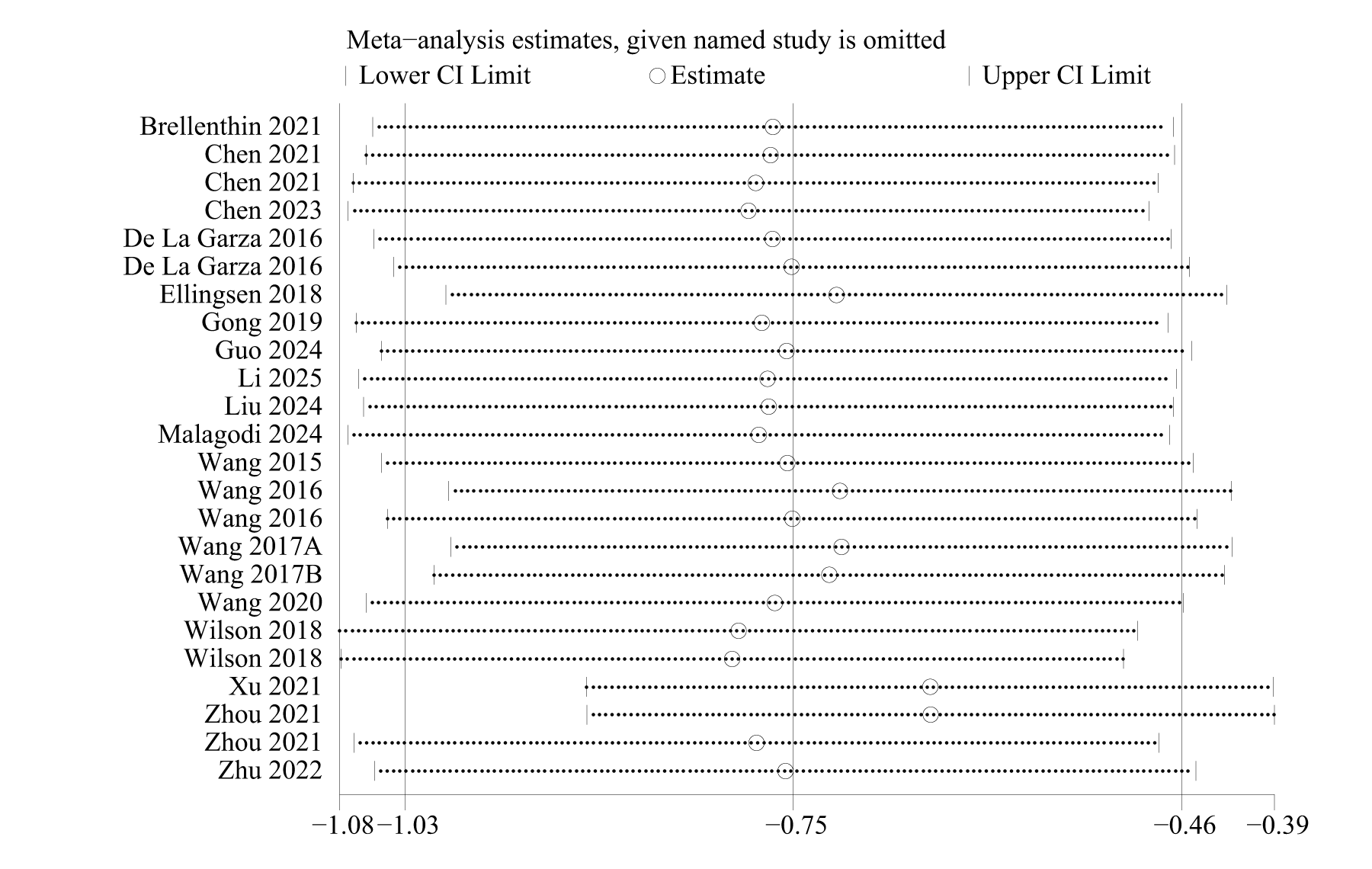
**

**Figure 5: Subgroup analysis of the effect of an aerobic exercise intervention on the effect of drug craving in subjects (model after exclusion of high sensitivity studies)**

**
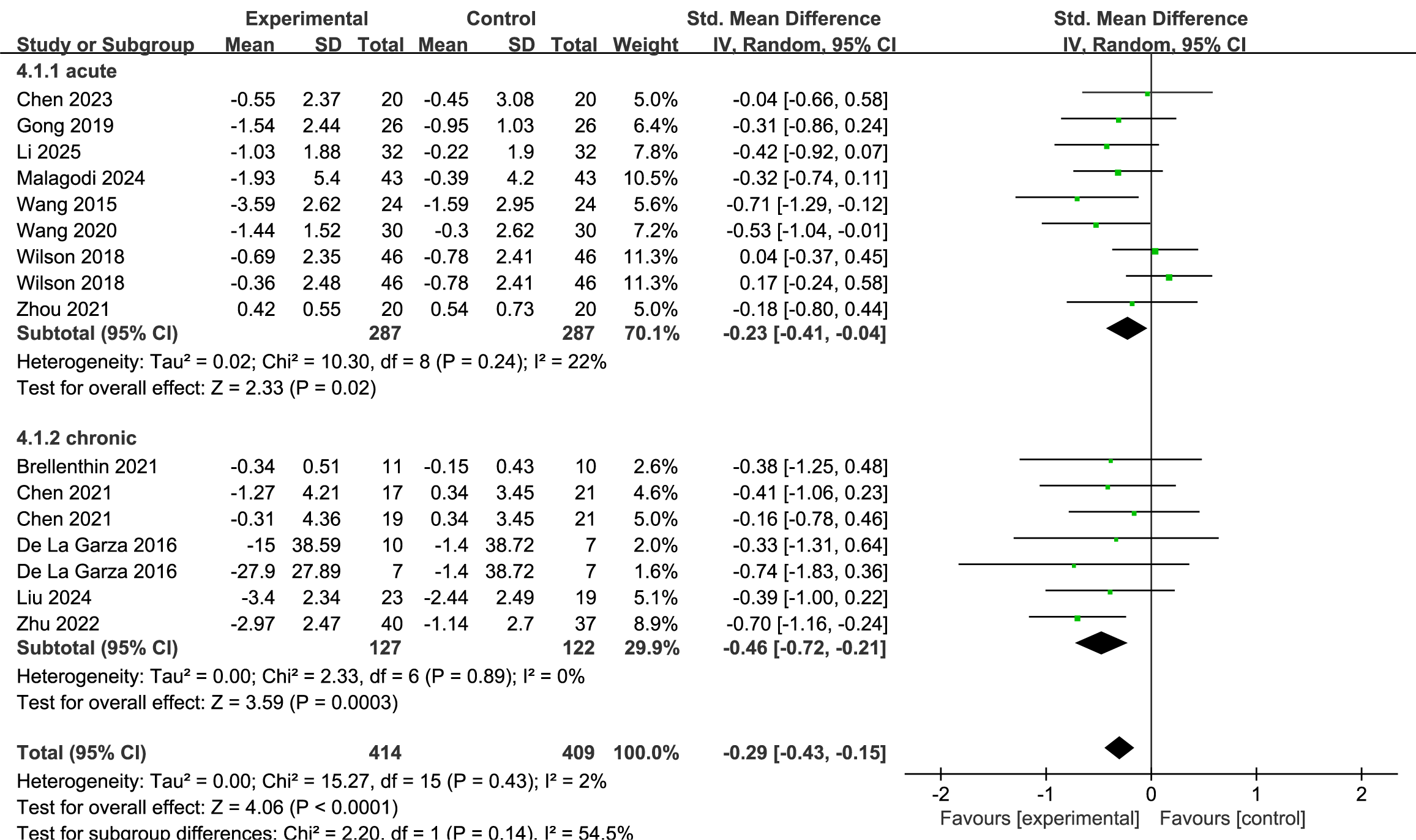
**

**Figure 6: Subgroup analysis of the effect of traditional Chinese exercise intervention on the effect of drug cravings in subjects**

**
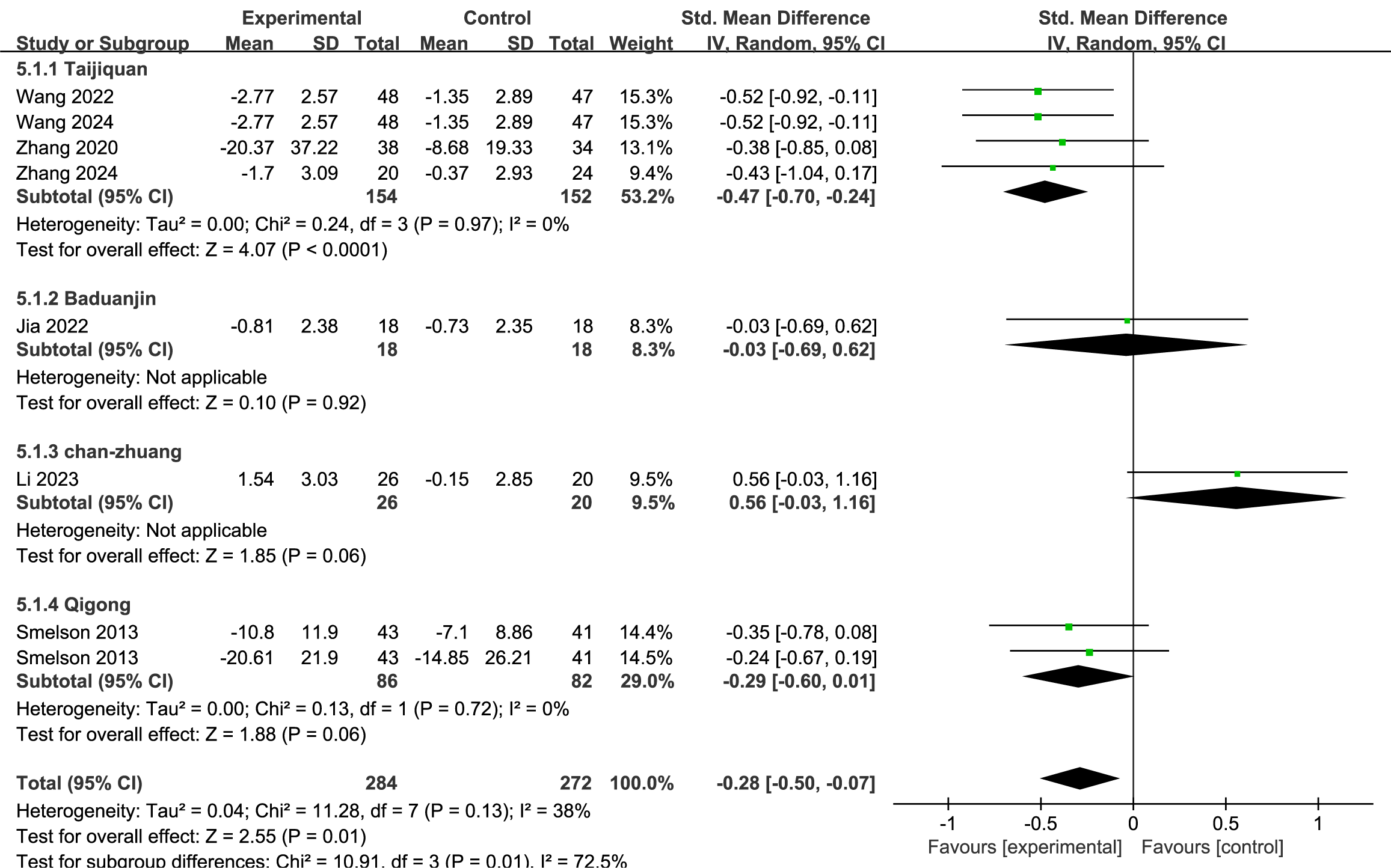
**

**2 .Dose-response analysis of exercise interventions**

**2.1 Examining network Connectivity**

**
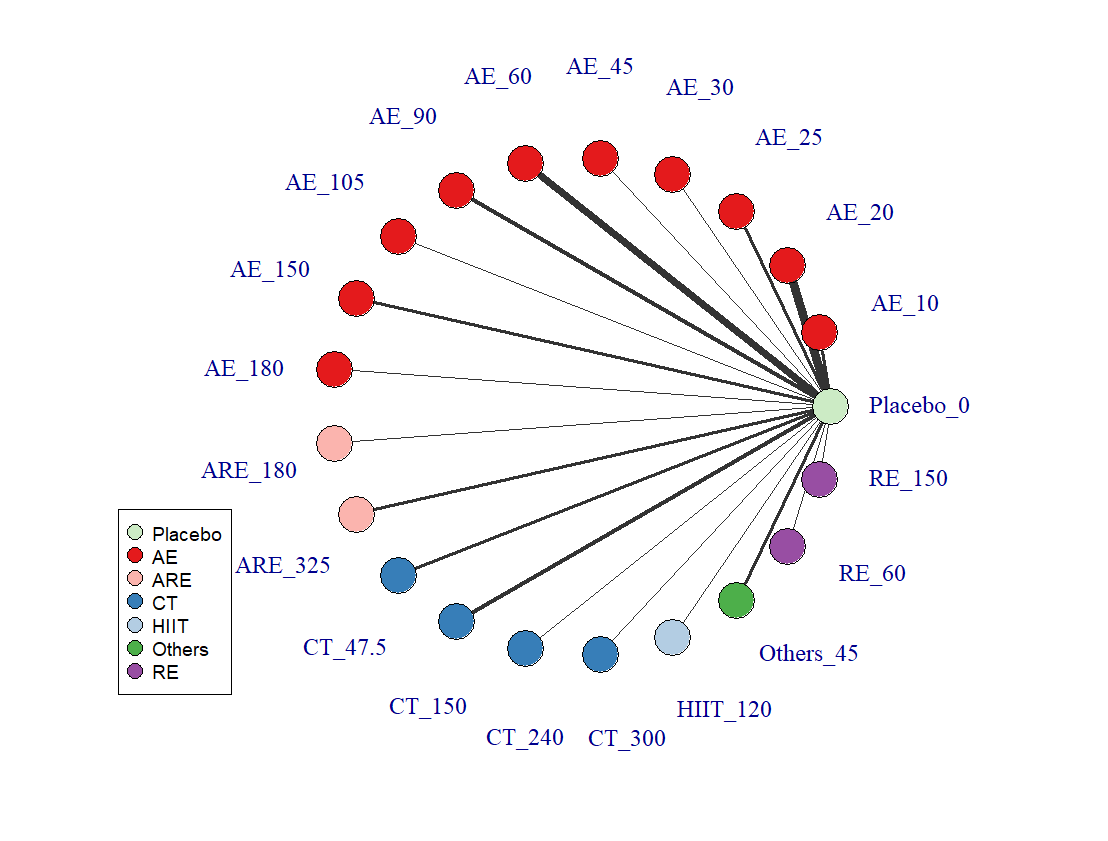
**

**2.2 Examining the dose-response relationship**

2.2.1 Total Exercise Dose Relationship

**
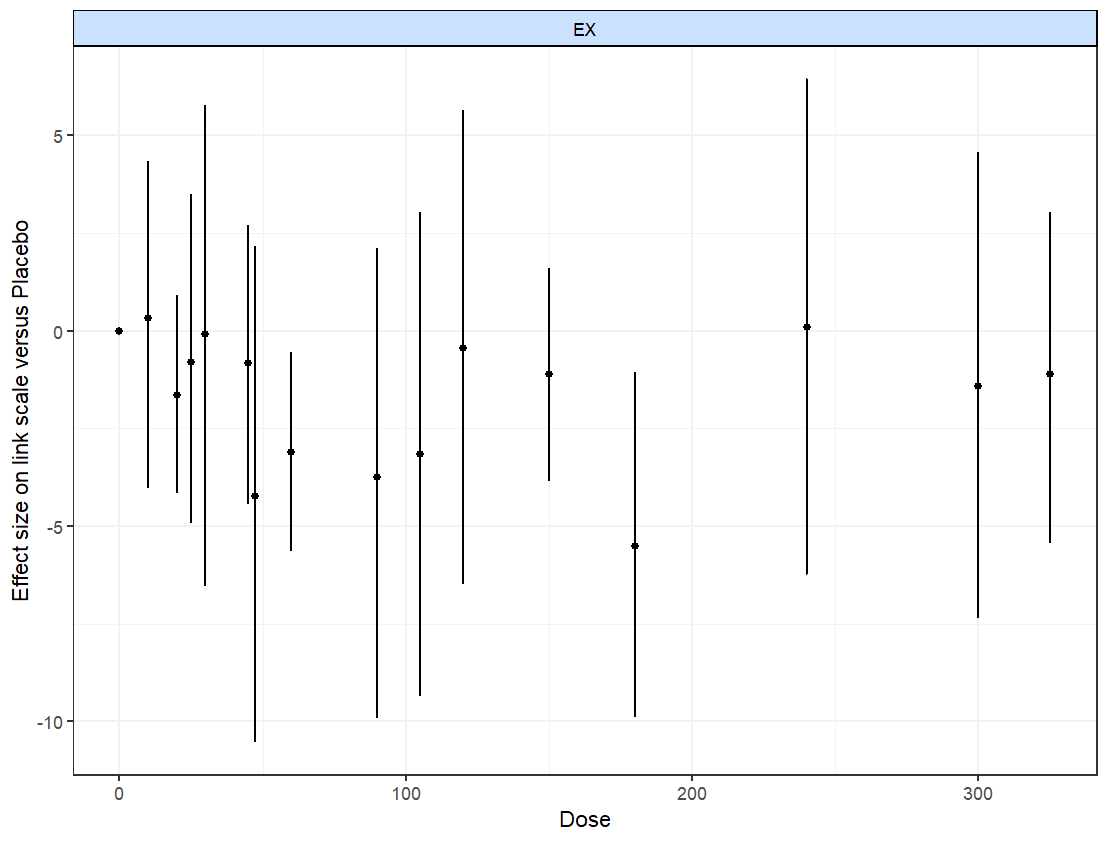
**

2.2.2 Individual Motion Measurement Relationships

**
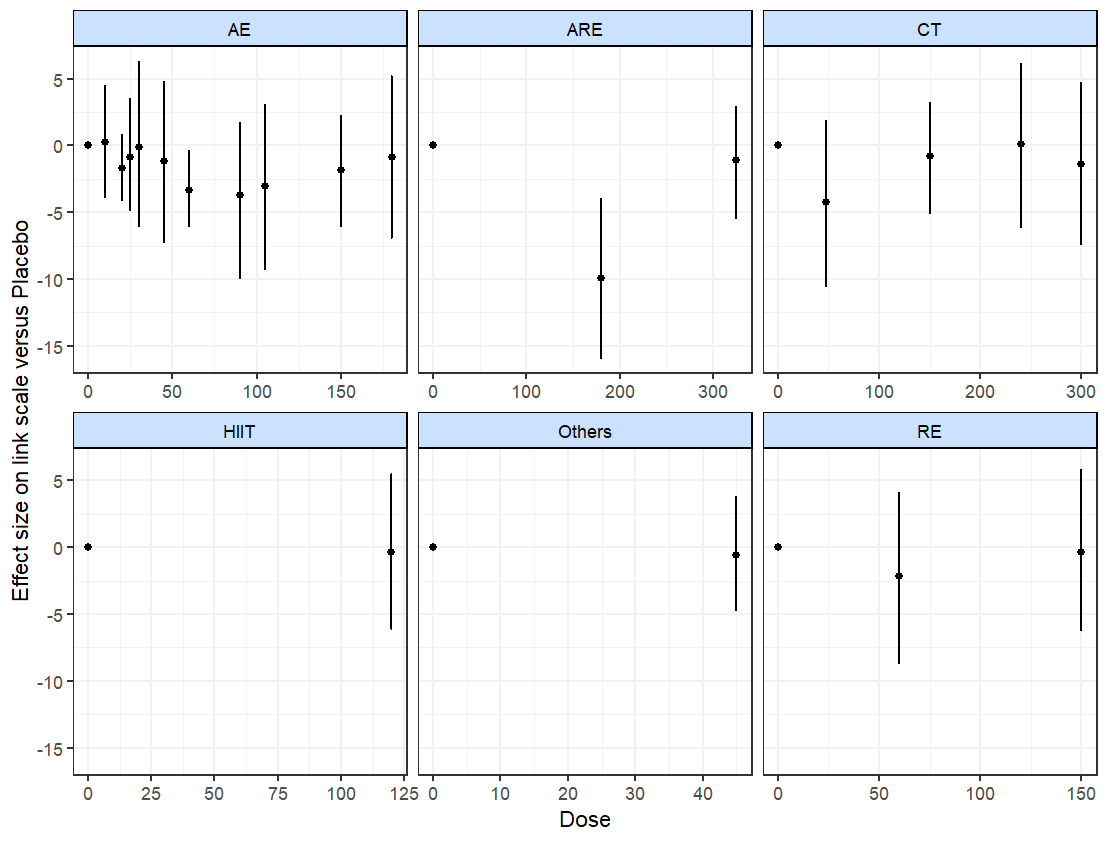
**

1. **Late estimates**
   1. **Deviation maps**
      1. Total Motion Deviation Chart


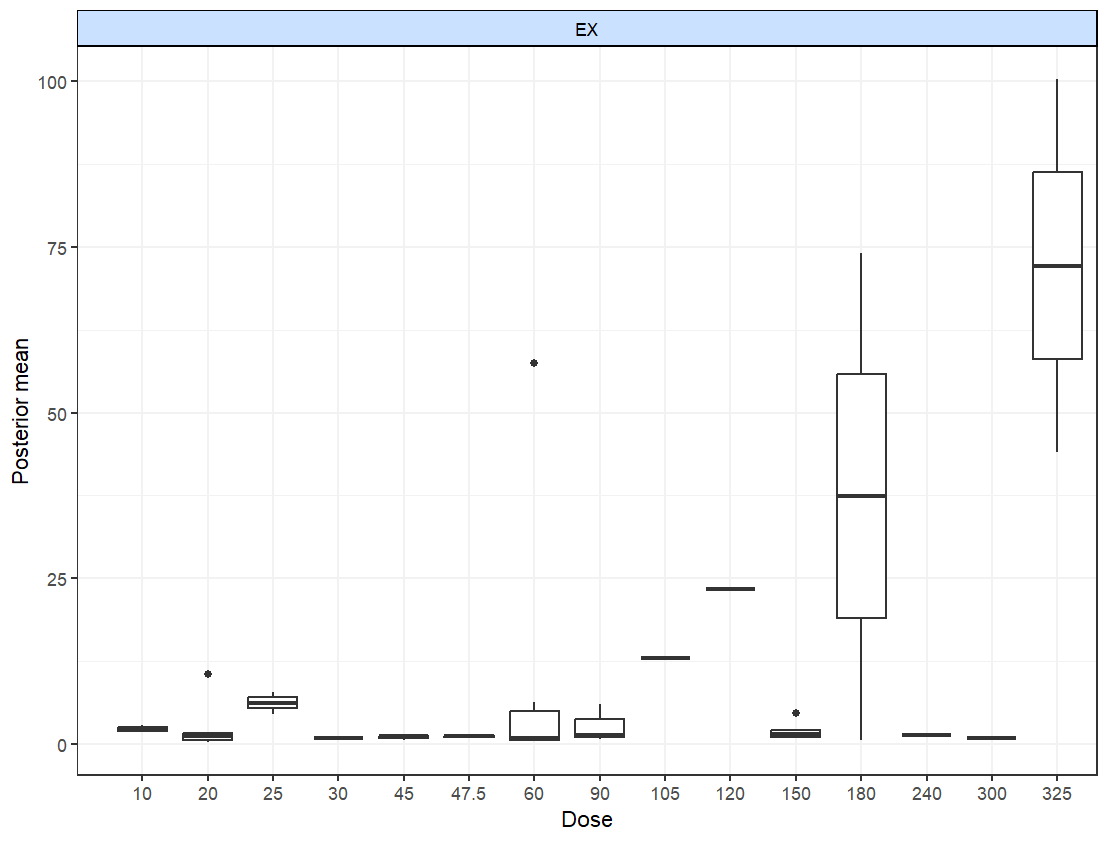


- - 1. Deviation charts for individual sports

**
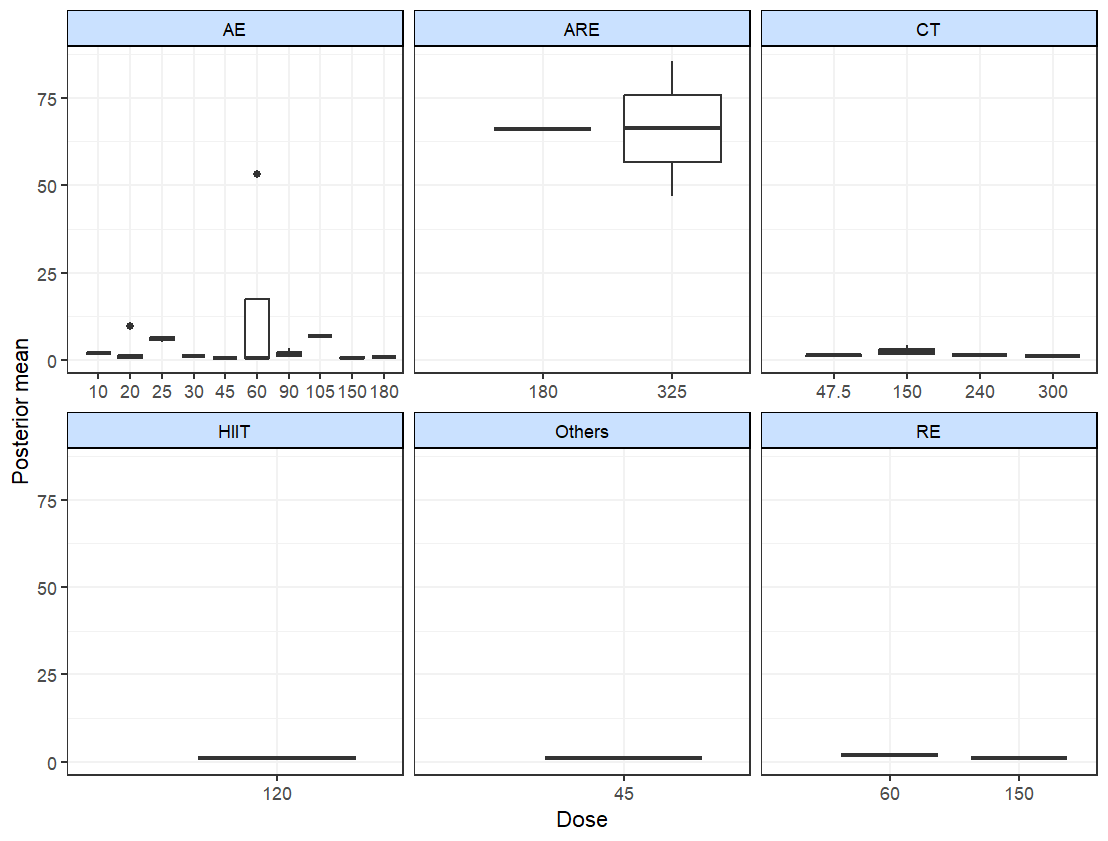
**

- 1. **Fitting value monitoring**
     1. Monitoring of total motion fit values


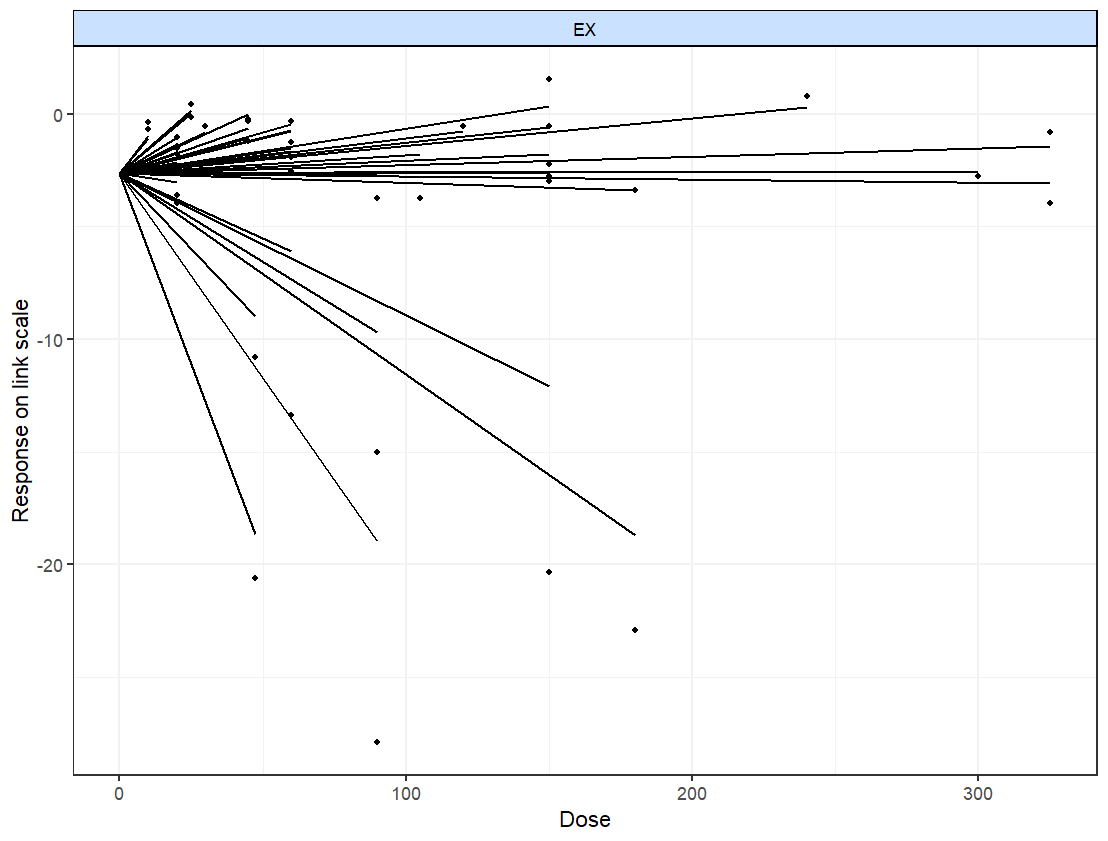


- - 1. Monitoring of the fitted values for each motion


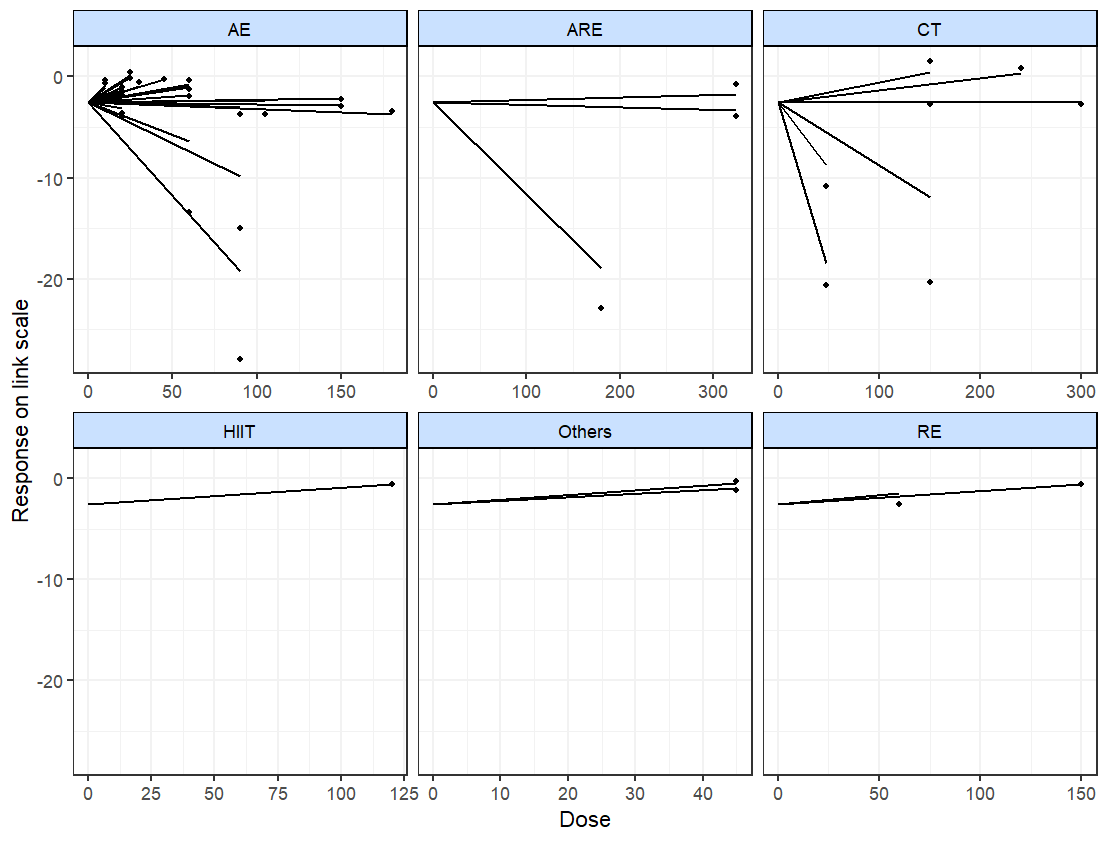


- 1. **Ranking**
     1. Ranking of the emax model for each exercise intervention


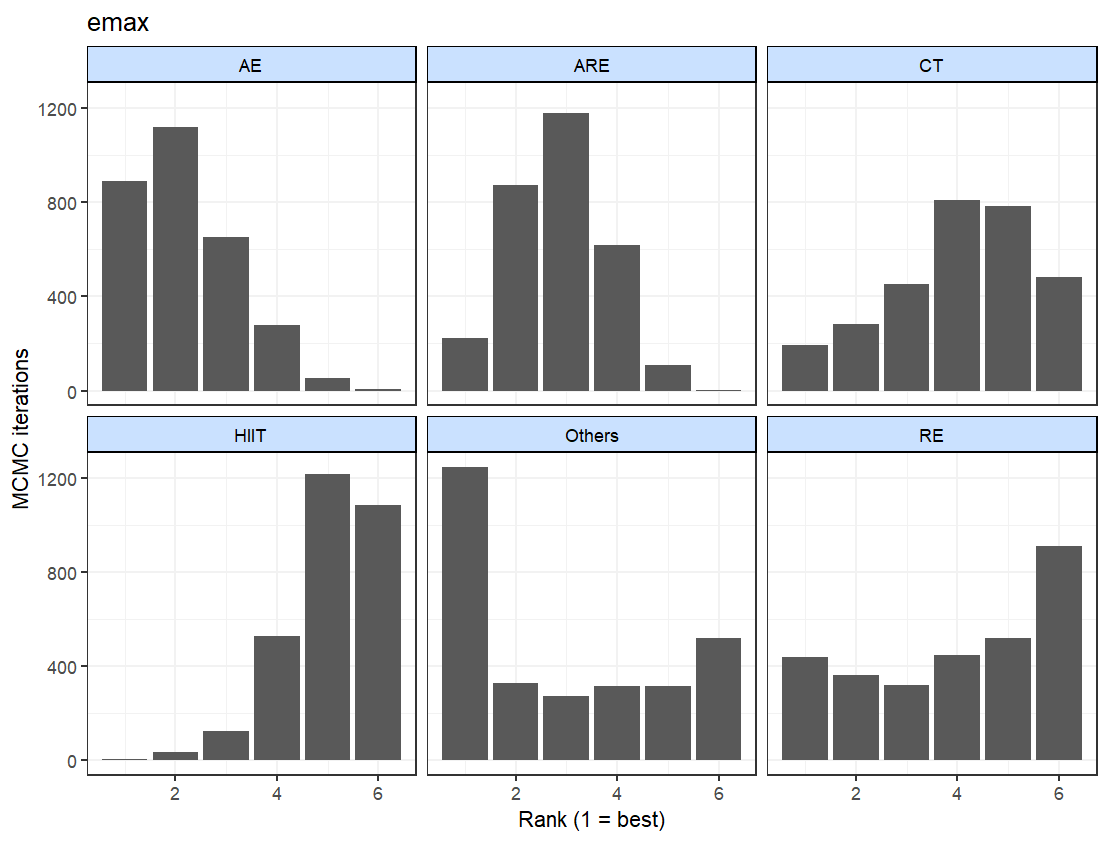


- - 1. Ranking of the ed50 model for each exercise intervention


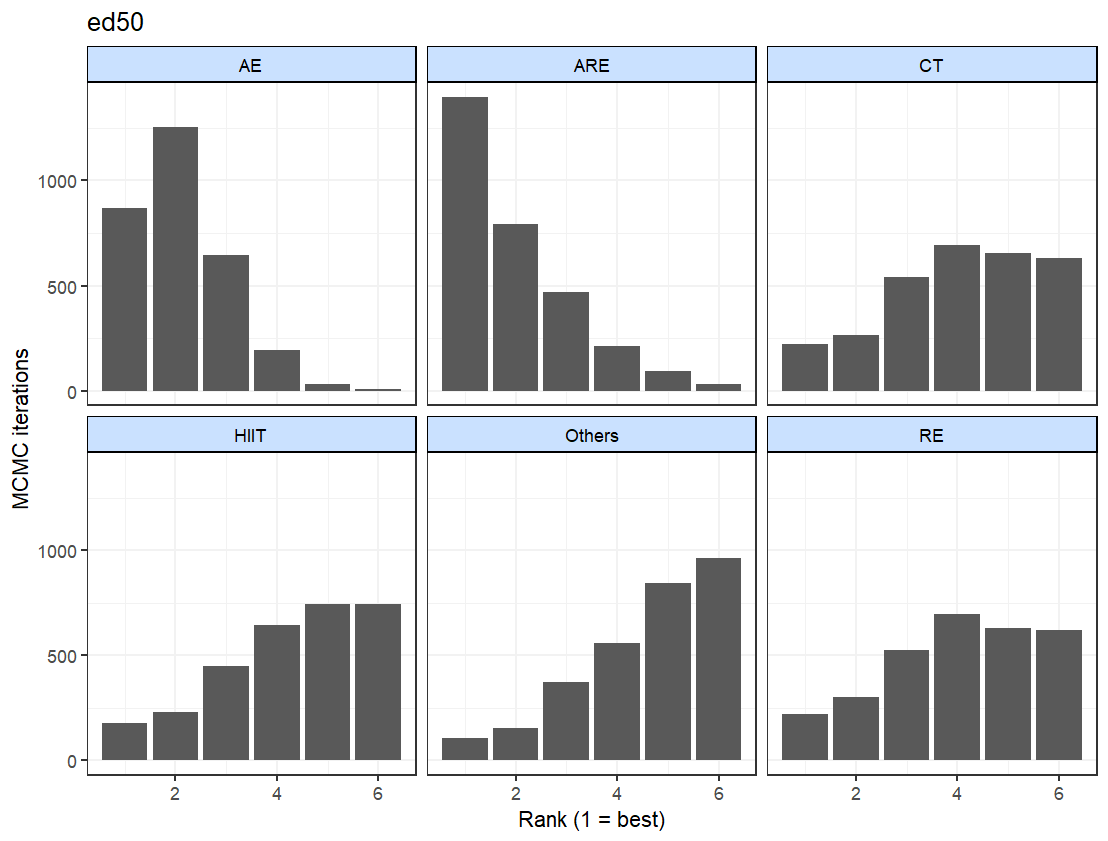


- - 1. Ranking curves based on two models


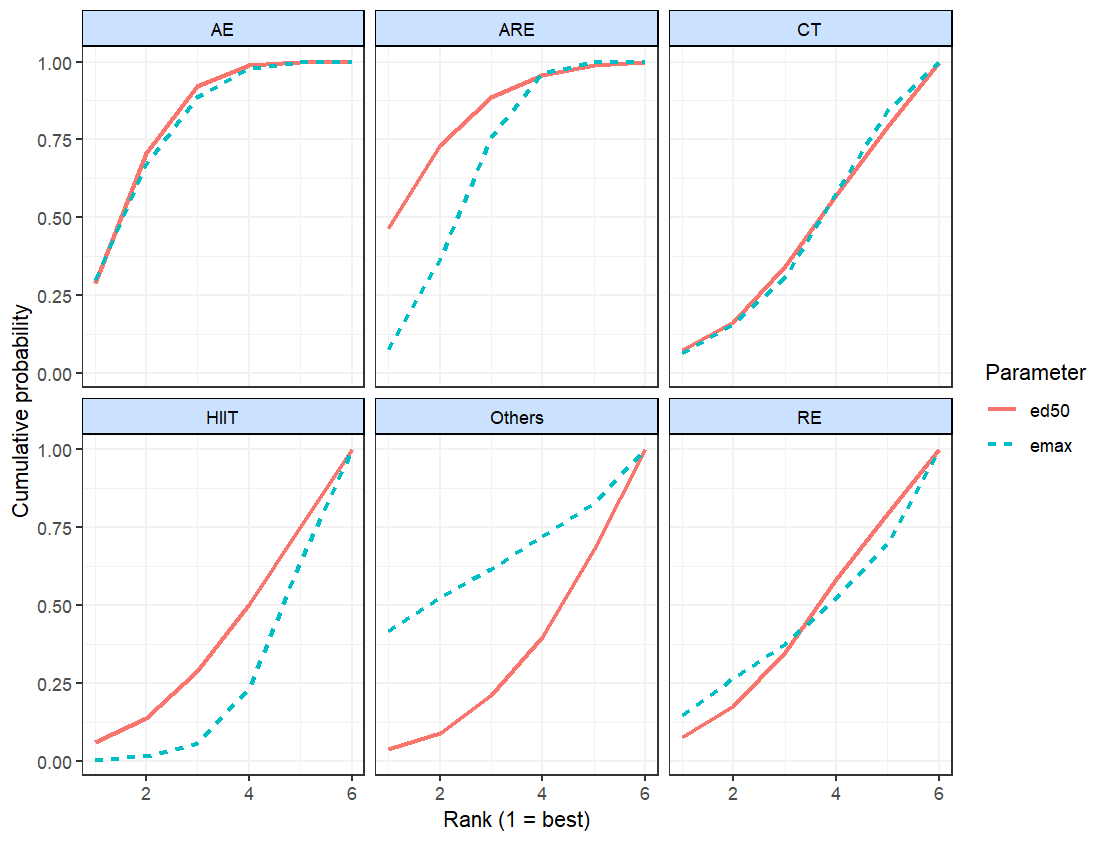


1. **Prediction**
   1. **Predictive effect curves**

4.1.1 Prediction curves for total exercise dose


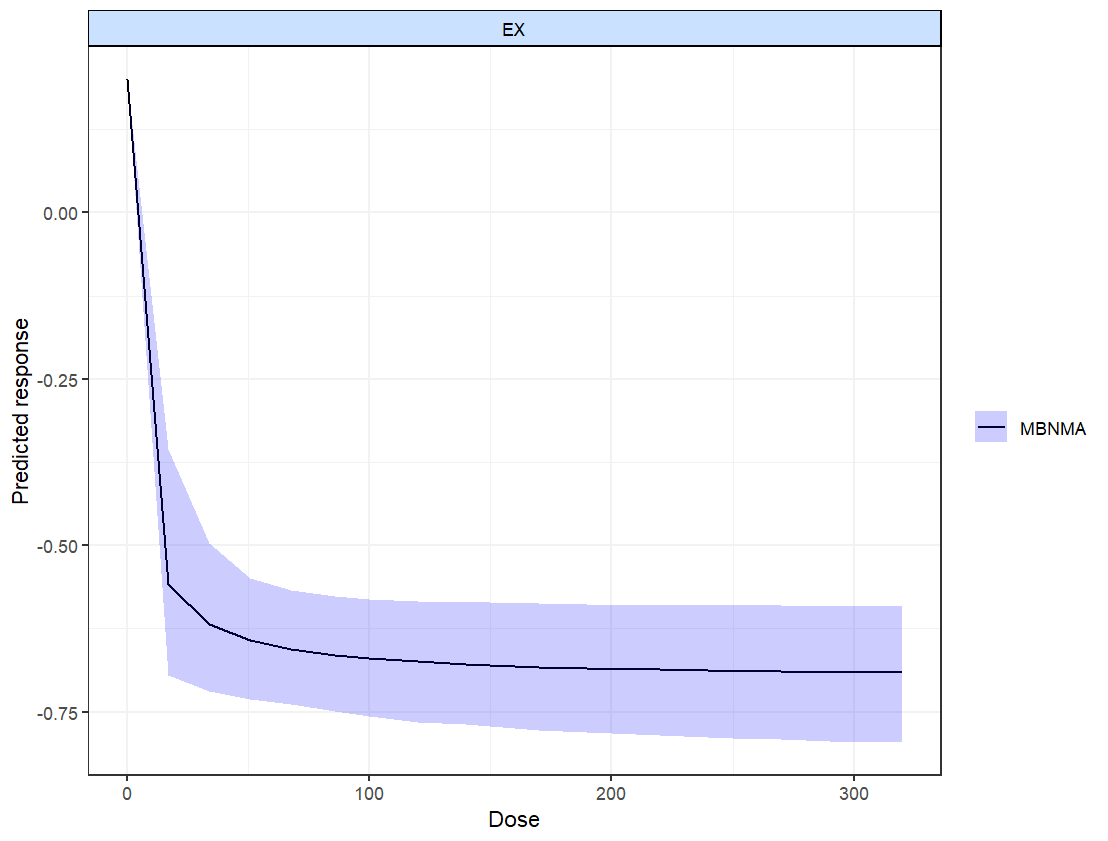


4.1.2 Prediction curves for the effects of each exercise intervention


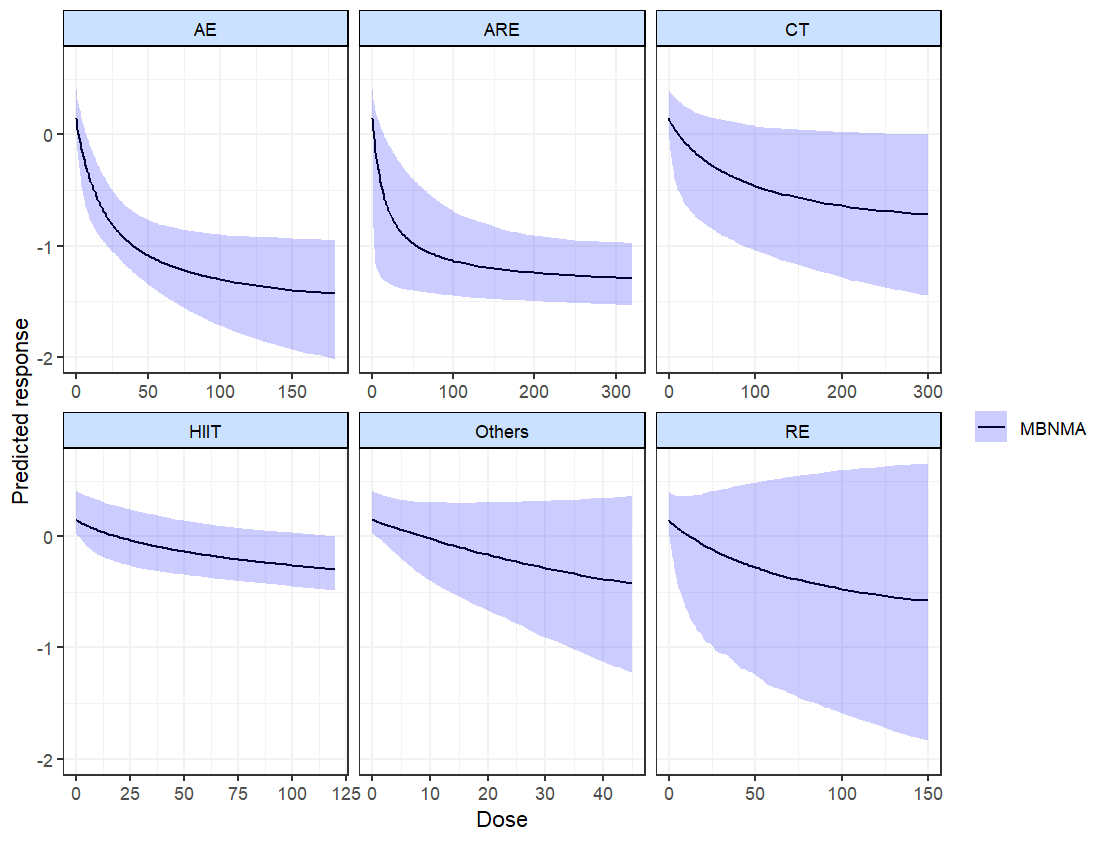


- 1. **Predictive effect curves with specific effect intervals**


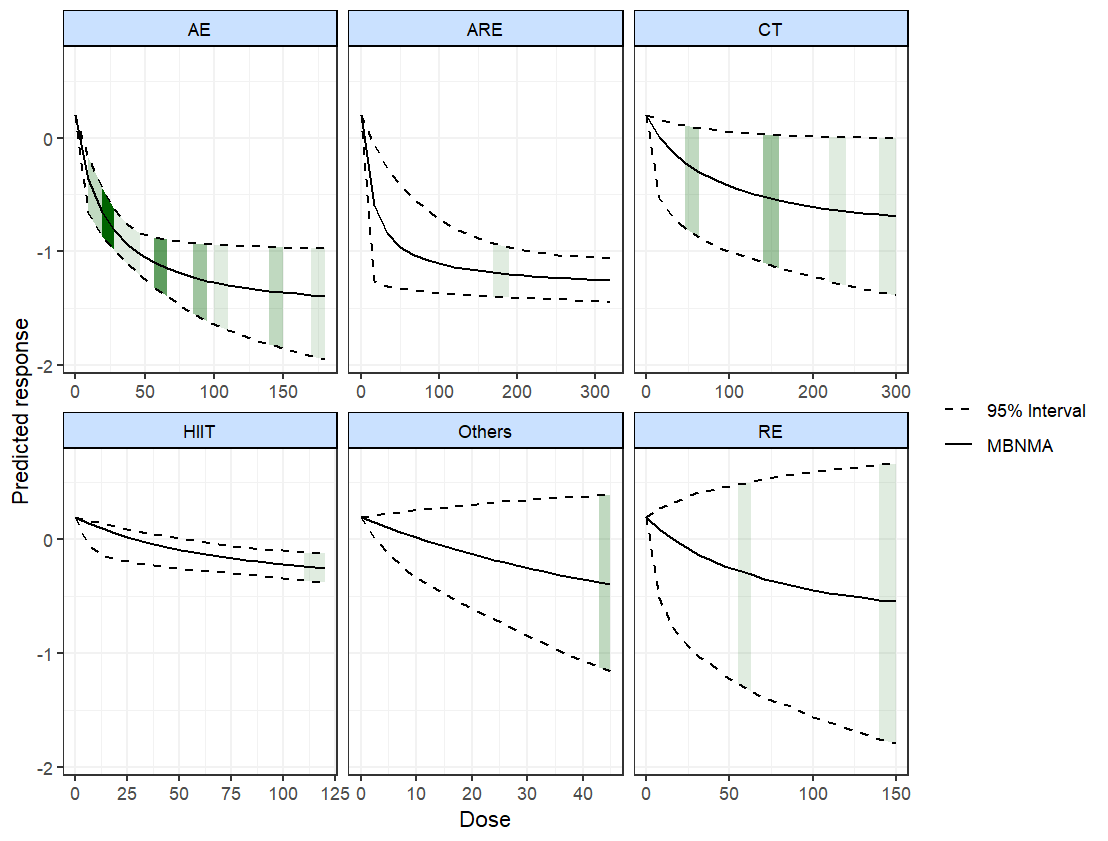


**5. Ranking predicted response**

**5.1 Overall projections**


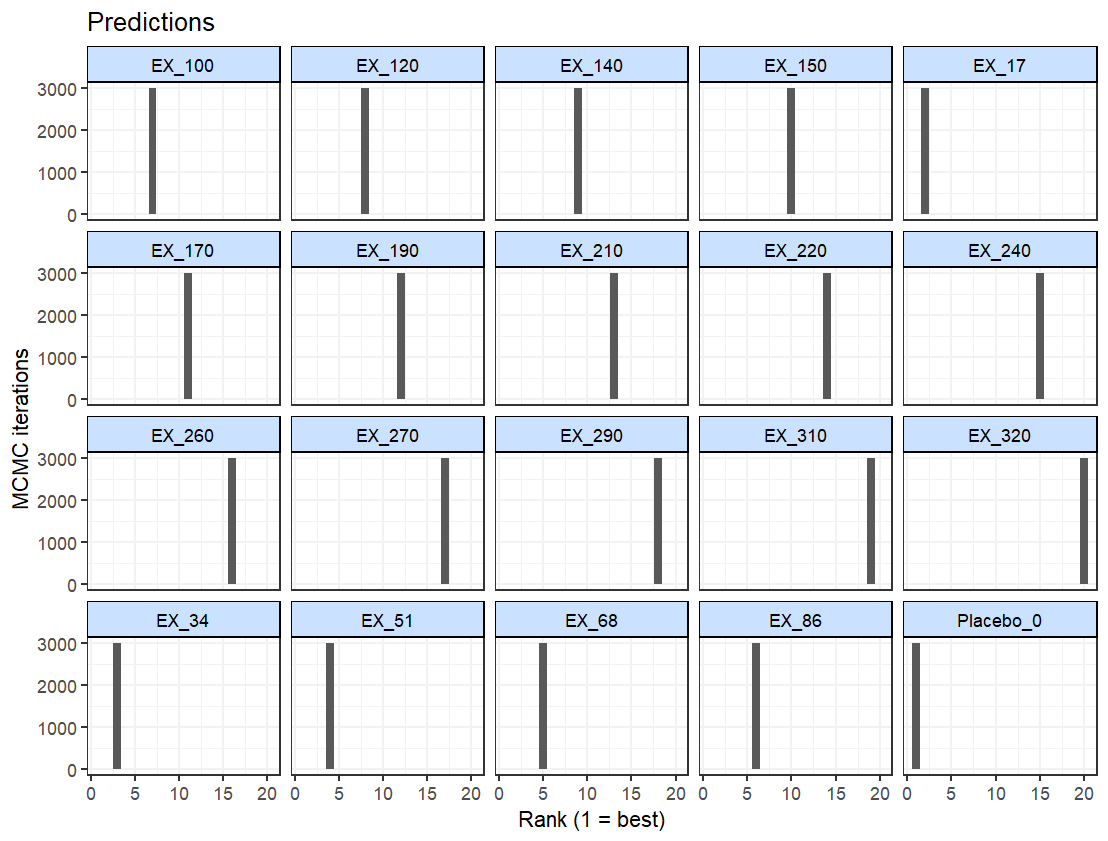


**5.2 Projections**


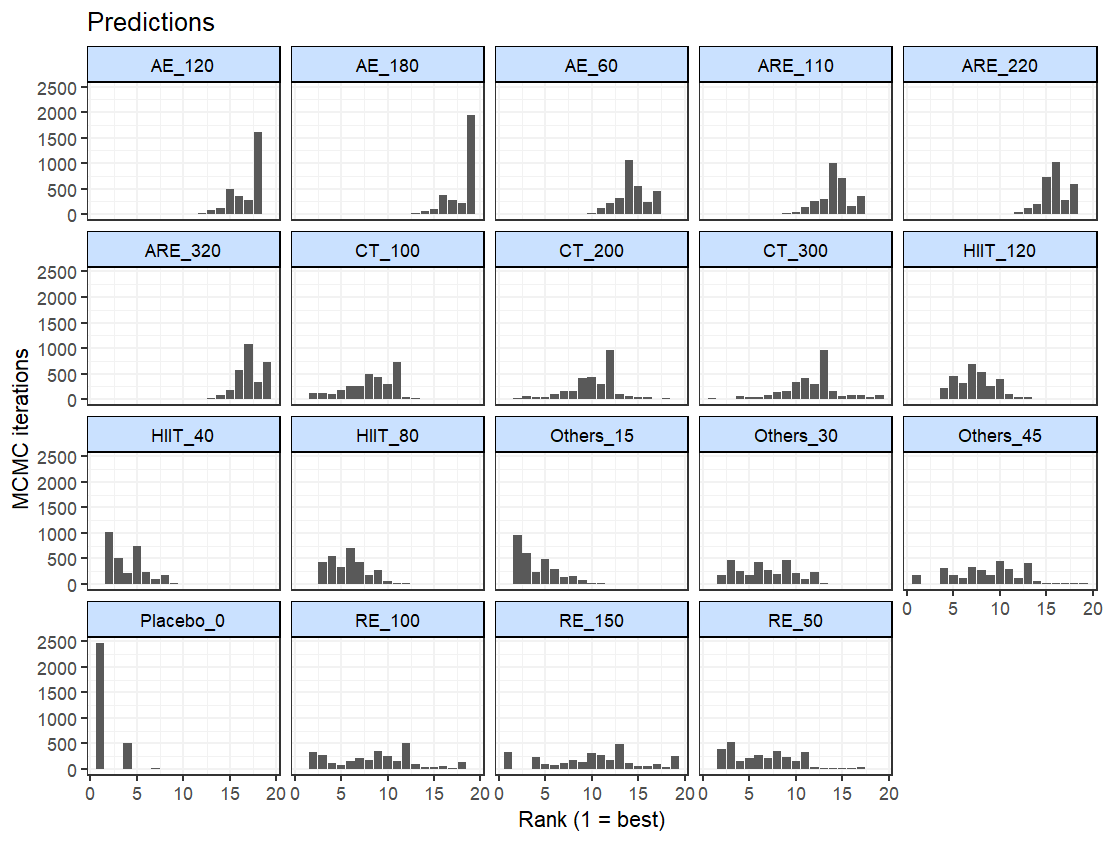


**6. Consistency Testing**


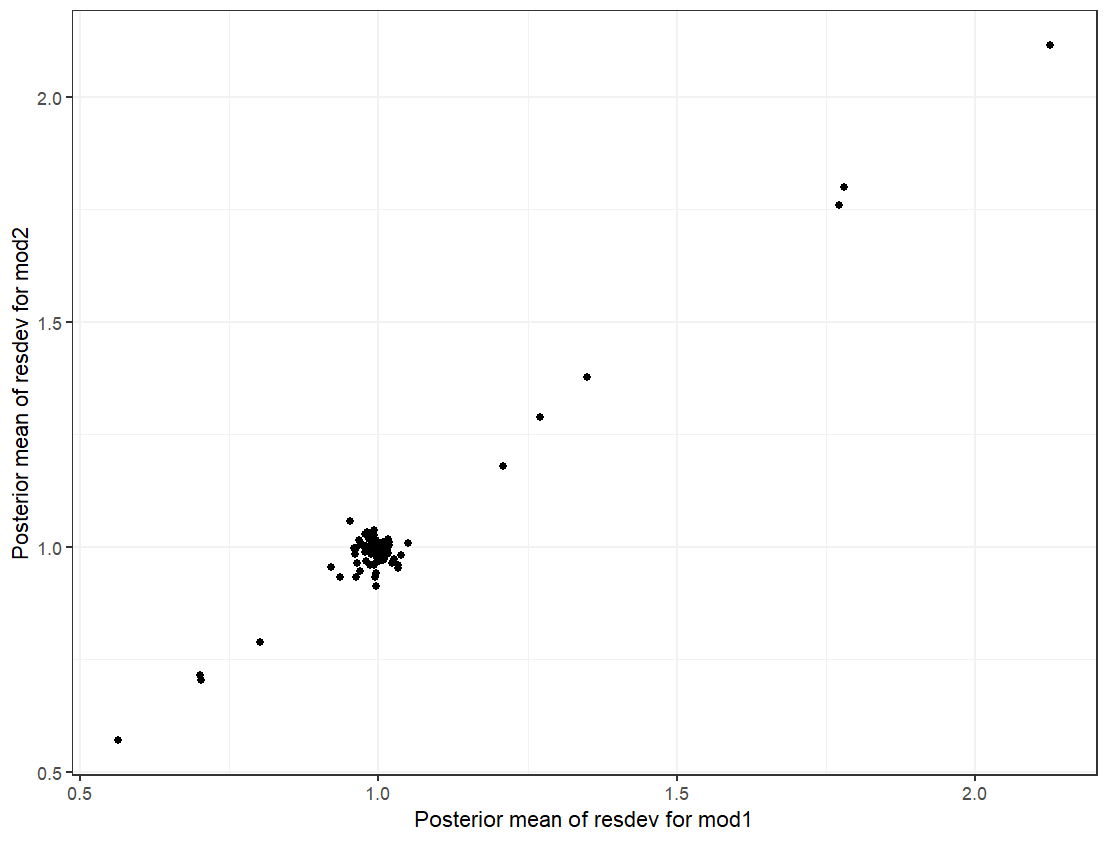


1. **Reporting bias and GRADE grade quality**
   1. **Risk of bias**

**
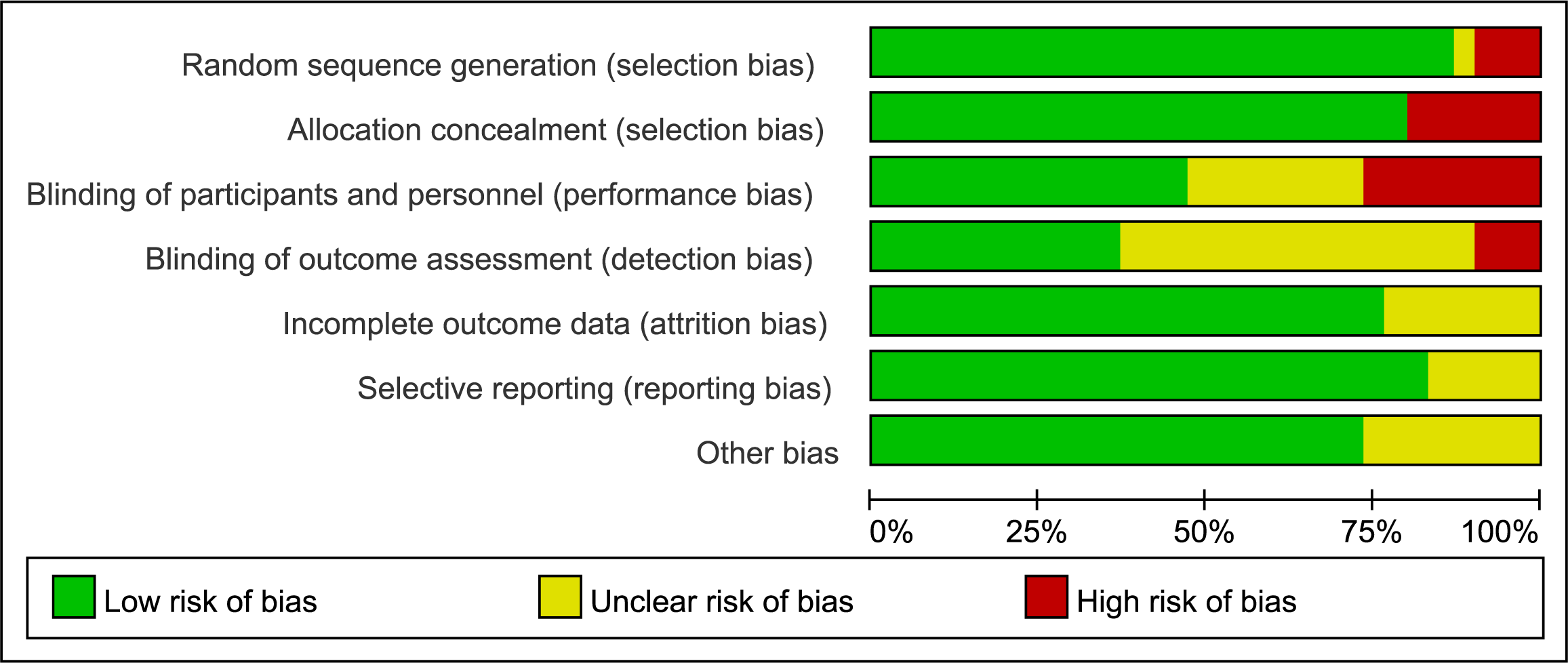

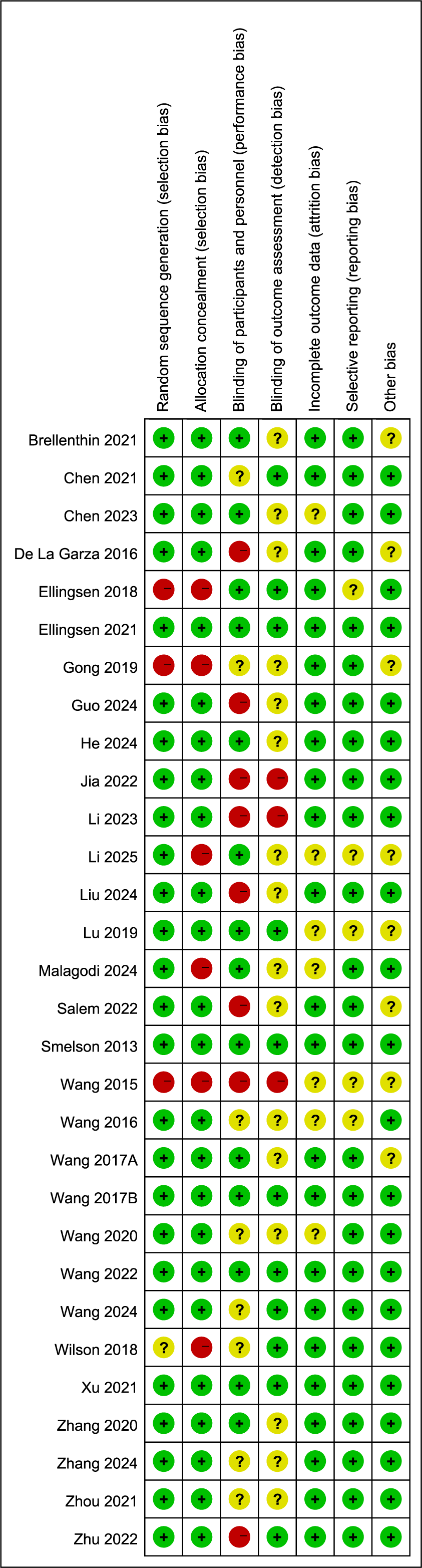
**

- 1. **GRADE summary of included studies**

We used the GRADE method to determine the quality of the research evidence. Due to the limitations of the included literature, we only performed a quality assessment of the included randomized controlled trials. The GRADE rating is reduced from 'high', and 'low' to 'very low' in the following cases.

1. Risk of bias: The included literature was assessed using the Cochrane Handbook and a risk assessment tool. If more than 50% of the included studies have an 'uncertain risk' or 'high risk' in the overall assessment, there is a risk of bias.

2. Directness: The study population, intervention, and outcome of the randomized controlled trial are assessed to determine whether they are directly relevant to the purpose of this systematic review.

3. Publication bias: The included randomized controlled trials are assessed for publication bias and potential conflicts of interest.

4. Publication precision: The 95% confidence intervals of the experimental results of the included randomized controlled trials are checked in turn. A valid interval is one in which the 95% confidence interval does not include 0.

5. Sample size: Determine whether the sample size of the randomized controlled trial is sufficient to influence the statistical results of the randomized controlled trial. The current study defined a total study population greater than 20 as not being at risk for sample size.

| Table 2: GRADE summary | | | | | | |
| --- | --- | --- | --- | --- | --- | --- |
| Author(years) | Risk of bias | Directness | Publication bias | Publication precision | Sample size | Overall |
| Smelson(2013) | + | + | + | + | + | High |
| Wang(2015) | - | + | - | + | + | Very low |
| Wang(2016) | + | + | - | + | + | Low |
| De La Garza (2016) | _ | + | + | + | + | Low |
| Wang(2017)A | + | + | - | + | + | Low |
| Wang(2017)B | + | + | - | + | + | Low |
| Ellingsen(2018) | - | + | - | + | - | Very low |
| Wilson(2018) | - | + | + | + | + | Low |
| Lu(2019) | + | + | - | + | + | Low |
| Gong(2019) | - | + | + | + | + | Low |
| Wang(2020) | - | + | + | + | + | Low |
| Zhang(2020) | - | + | + | + | + | Low |
| Brellenthin(2021) | - | + | + | + | + | Low |
| Chen(2021) | - | + | + | + | + | Low |
| Ellingsen(2021) | + | + | + | + | + | High |
| Xu(2021) | + | + | - | + | + | Low |
| Zhou(2021) | - | + | - | + | + | Very low |
| Salem(2022) | - | + | + | + | + | Low |
| Wang(2022) | + | + | + | + | + | High |
| Zhu(2022) | - | + | + | + | + | Low |
| Jia(2022) | - | + | + | + | + | Low |
| Chen(2023) | - | + | + | + | + | Low |
| Li(2023) | - | + | + | + | + | Low |
| Guo(2024) | - | + | + | + | + | Low |
| Malagodi(2024) | - | + | + | + | + | Low |
| Wang(2024) | - | + | + | + | + | Low |
| Zhang(2024) | - | + | + | + | + | Low |
| He(2024) | - | + | - | + | + | Very low |
| Liu(2024) | - | + | + | + | + | Low |
| Li(2025) | - | + | + | + | + | Low |

**8 Publication Bias**

**8.1 Aerobic exercise publication bias**


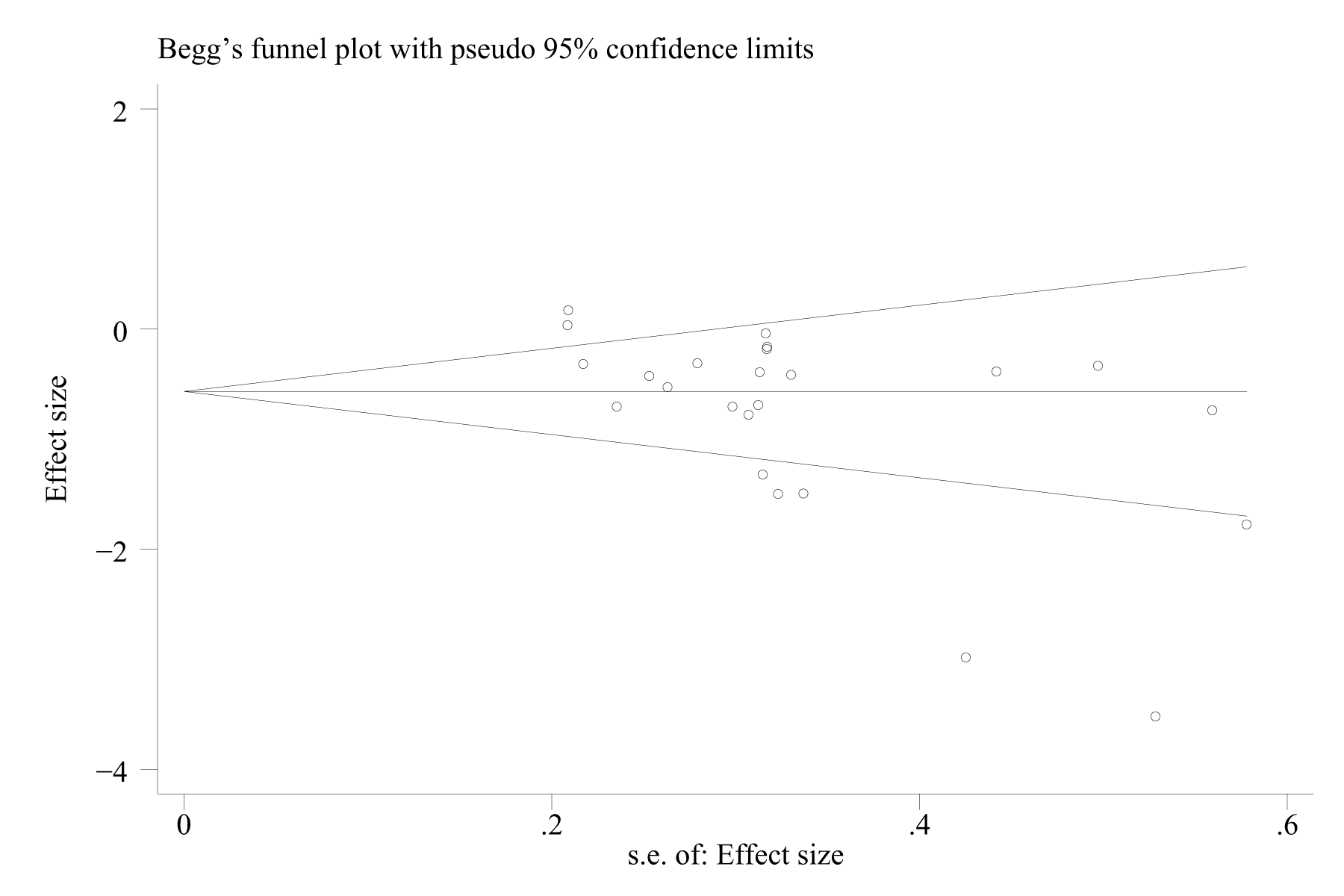


**8.2 Traditional campaign publication bias**


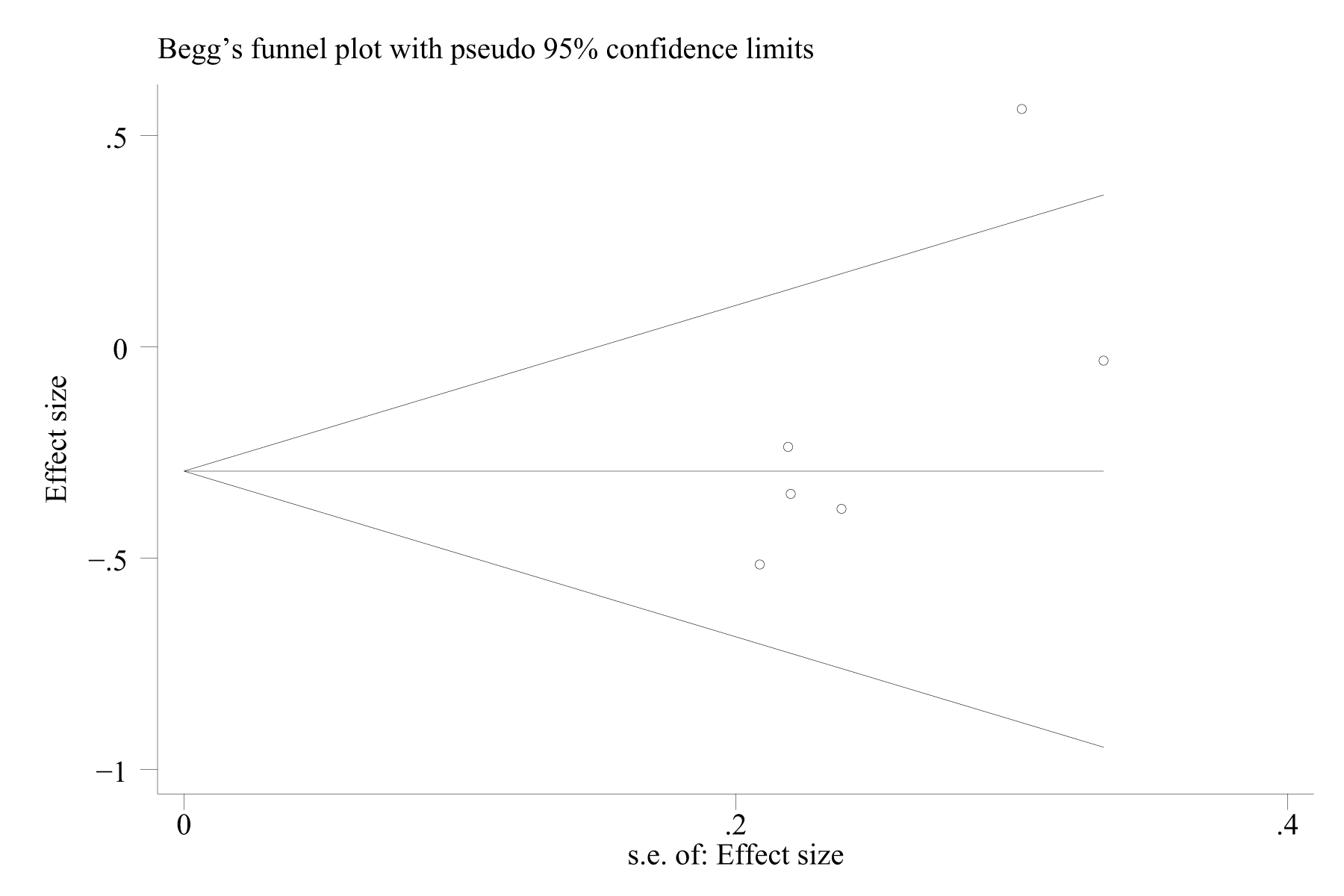


**9. Search example**

| Database | Retrieval Strategy | Search Date | Search results |
| --- | --- | --- | --- |
| Pubmed | Search: ****("drug" or "substance use disorder" or "cannabis" or "cocaine" or "methamphetamine") and ("exercise" or "training" or "physical activity" or "sports") and ("craving" or "VAS").****  ("drug"[All Fields] OR "substance use disorder"[All Fields] OR "cannabis"[All Fields] OR "cocaine"[All Fields] OR "methamphetamine"[All Fields]) AND ("exercise"[All Fields] OR "training"[All Fields] OR "physical activity"[All Fields] OR "sports"[All Fields]) AND ("craving"[All Fields] OR "VAS"[All Fields]) | February 2025 | 1437 |
| Web of science | **("drug" or "substance use disorder" or "cannabis" or "cocaine" or "methamphetamine") and ("exercise" or "training" or "physical activity" or "sports") and ("craving" or "VAS").** | February 2025 | 1320 |
| EMBASE | **("drug" or "substance use disorder" or "cannabis" or "cocaine" or "methamphetamine") and ("exercise" or "training" or "physical activity" or "sports") and ("craving" or "VAS").** | February 2025 | 988 |
| CNKI | “成瘾与运动”，“药物成瘾与训练”，“成瘾与渴求度”，“甲基苯丙胺与运动”. | February 2025 | 583 |
